# A combination of genome‐wide and transcriptome‐wide association studies reveals genetic elements leading to male sterility during high temperature stress in cotton

**DOI:** 10.1111/nph.17325

**Published:** 2021-05-02

**Authors:** Yizan Ma, Ling Min, Junduo Wang, Yaoyao Li, Yuanlong Wu, Qin Hu, Yuanhao Ding, Maojun Wang, Yajun Liang, Zhaolong Gong, Sai Xie, Xiaojun Su, Chaozhi Wang, Yunlong Zhao, Qidi Fang, Yanlong Li, Huabin Chi, Miao Chen, Aamir Hamid Khan, Keith Lindsey, Longfu Zhu, Xueyuan Li, Xianlong Zhang

**Affiliations:** ^1^ National Key Laboratory of Crop Genetic Improvement Huazhong Agricultural University Wuhan 430070 China; ^2^ Xinjiang Academy of Agricultural Science Xinjiang 830000 China; ^3^ Department of Biosciences Durham University Durham DH1 3LE UK

**Keywords:** anther development, eQTL, *Gossypium hirsutum*, high temperature, population genetics, transcriptome analysis

## Abstract

- Global warming has reduced the productivity of many field‐grown crops, as the effects of high temperatures can lead to male sterility in such plants. Genetic regulation of the high temperature (HT) response in the major crop cotton is poorly understood.
- We determined the functionality and transcriptomes of the anthers of 218 cotton accessions grown under HT stress. By analyzing transcriptome divergence and implementing a genome‐wide association study (GWAS), we identified three thermal tolerance associated loci which contained 75 protein coding genes and 27 long noncoding RNAs, and provided expression quantitative trait loci (eQTLs) for 13 132 transcripts.
- A transcriptome‐wide association study (TWAS) confirmed six causal elements for the HT response (three genes overlapped with the GWAS results) which are involved in protein kinase activity. The most susceptible gene, *GhHRK1*, was confirmed to be a previously uncharacterized negative regulator of the HT response in both cotton and Arabidopsis.
- These functional variants provide a new understanding of the genetic basis for HT tolerance in male reproductive organs.

## Introduction

Global warming has raised the average global temperature by *c*. 1.5°C since the beginning of the 20^th^ century and has led to unpredictable high temperatures (HTs) (Seneviratne *et al.,* 2018). Although the yields of many crops have increased in recent decades, sensitivity to environmental stresses has also increased, which is problematic as the climate continues to change (Lobell *et al.,* 2014). Major crops are reported to be vulnerable to the impacts of climate change (Schauberger *et al.,* 2017; Zhao *et al.,* 2017). Cotton is cultivated globally for the production of its natural fibers, and it is commonly affected by HTs. Several reports indicated that HT stress could lead to male sterility in cotton, which results in substantive yield losses (Pettigrew, 2008; Min *et al.,* 2014; Zahid *et al.,* 2016). Thus, breeding cotton germplasm with HT tolerance and understanding the mechanisms involved in the HT response in male reproductive organs is necessary.

In flowering plants, the male reproductive development process involves male meiosis (tetrad stage), microspore development (tapetum degradation stage) and anther dehiscence (anther dehiscence stage) (Ma, 2005). High temperature stress influences the male reproductive organs in particular, resulting in abnormal anther structure and impaired pollen viability. Recent research has uncovered the molecular basis of the HT response in male reproductive organs. Genes which regulate energy substances such as lipids and sugars have been found to be essential for the HT response in Arabidopsis, maize and cotton. The disordered accumulation of energy substances during the tetrad stage of anthers usually results in male sterility under HT stress (Begcy *et al.,* 2019; Khan *et al.,* 2020; Zhu *et al.,* 2020). Accumulated auxin has been shown to mitigate pollen sterility in Arabidopsis and wheat under HT conditions (Sakata *et al.,* 2010; Abbas *et al.,* 2018). However, excessive auxin leads to male sterility in cotton anthers under HT stress, indicating a distinct regulatory role of plant hormones in various species (Min *et al.,* 2014; Ding *et al.,* 2017). Furthermore, disrupted epigenetic modifications involving small RNA, DNA methylation and tRNA modification could disorder gene expressions and lead to male sterility in maize, cotton and rice (Y. Ma *et al.,* 2018; Chen *et al.,* 2019; Teng *et al.,* 2020; Xu *et al.,* 2020). These findings imply that disturbed gene expression in the early developmental stages of male reproductive organs could lead to pollen sterility.

Several studies have elucidated heat‐response pathways and related metabolic networks, but the genetic architecture of complex stress response traits in agriculturally important crops is much less well understood. By implementing genomic variation maps and advanced bioinformatic strategies, genome‐wide association studies (GWASs) based on linkage disequilibrium (LD) have produced remarkable advances in the genetic dissection of complex traits through the use of efficient algorithms. An association study can analyze genetic correlations between single nucleotide polymorphism (SNP) markers and noted phenotypes to provide candidate regions in the genome. Subsequent LD analysis of candidate intervals allows high resolution QTL mapping and causal gene identification and cloning. Over the past decade, the genetic basis of a large number of traits has been determined in major crops (Li *et al.,* 2013; Mao *et al.,* 2015; Zhou *et al.,* 2015; Wang *et al.,* 2017; Z. Ma *et al.,* 2018). A comprehensive GWAS has identified 4820 genes contributing to 13 fiber‐related traits in cotton, providing novel genetic resources for fiber improvement (Z. Ma *et al.,* 2018). By combining the GWAS technique and pairwise LD analysis, it has been confirmed that an F‐box protein (ZmFBL41) confers resistance to banded leaf and sheath blight in maize (Li *et al.,* 2019). Meanwhile, parallel transcriptome sequencing provides an expression profile of each gene as well as comprehensive variations in the gene coding regions. These genetic resources facilitate the identification of eQTLs which could regulate the expression of genes. Thus far, eQTL regulatory maps have been successfully constructed in rice (Wang *et al.,* 2010; Kuroha *et al.,* 2017), maize (Fu *et al.,* 2013; Liu *et al*., 2017, 2020; Wang *et al.,* 2018), lettuce (Zhang *et al.,* 2017), tomato (Ranjan *et al.,* 2016) and cotton fiber (Li *et al.,* 2020). These eQTL maps uncover the critical roles of genomic variation in the regulation of gene expression. Furthermore, eQTLs can be used to estimate the effects on gene expression and then be combined with physical phenotypes to conduct transcriptome‐wide association studies (TWASs) to identify pivotal expression–trait associations (Gusev *et al.,* 2016). The TWAS algorithm has been successfully implemented in cotton fiber and rapeseed to identify causal genes for important agronomic traits (Li *et al.,* 2020; Tang *et al.,* 2020).

Cotton is mainly cultivated in summer, which means that cotton could be a useful object of study with which to research the mechanism of the HT response in male reproductive organs. Currently, the reasons for male sterility under HT stress in cotton require further elucidation. In order to uncover genetic regulatory mechanisms under HT stress in the anther, we performed massively parallel RNA sequencing (RNA‐seq) of anthers for a collection of 218 cotton accessions, cultivated for cotton production, providing abundant SNPs directly from transcriptionally active regions of the genome and potential genes for HT tolerance.

## Materials and Methods

### Determination of pollen viability

The population was planted in Alear, Xinjiang province in 2015; Turpan, Xinjiang province in 2016; and Wuhan, Hubei province in 2016 and 2017. In Wuhan, the sample panel was planted in two blocks at a field density of 27 000 plants ha^–1^ (Dai *et al.,* 2017). More than 12 individuals of each accession were planted in each row in each replicate. A thermometer was set in the field to show the highest and lowest daytime temperatures. High temperature (HT) stress was judged to occur when the daily maximum temperature was > 37°C for > 3 d. In both Alear and Turpan in Xinjiang province, two blocks were set in the field with a plant density of 195 000 ha^–1^. More than 30 individuals of each accession were set in each row. The thresholds for HT stress were the same as those used in Wuhan. Detailed field images and meteorological data are included in Supporting Information Fig. S1 and Table S1.

Flowers subjected to HT stress were harvested and immersed in 2,3,5‐Triphenyl tetrazolium chloride (TTC) solution (8 g TTC dissolved in 1 l phosphate buffer; pH = 7) to stain the pollen for viability. After staining for 1 h at room temperature, 2% (v/v) sulfuric acid solution was used to stop the staining reaction. Pollen grains were pipetted onto microscope slides and photographed using a Zeiss (Oberkochen, Germany) Axio Scope A1 microscope. At least three view fields were taken for each flower, and a total of > 50 flowers were harvested for TTC staining per sample. Pollen viability was measured as the ratio of normally stained pollen grains to total pollen counts. Phenotyping was accomplished following a multiple‐environment experiment which aimed to eliminate environmental effects. The broad sense heritability of pollen viability was estimated to be 0.937 (*H* ^2^). The best linear unbiased predictions (BLUPs) (Poland *et al.,* 2011) were used to evaluate pollen viability using the lme4 package in R.

### Sample preparation and RNA sequencing

All 218 accessions were cultivated in flat plots in a randomized block design group with two replicates in Alear, Xinjiang, China, in the summer of 2015. Based on findings from our previous study, cotton anthers underwent the tetrad stage while bud lengths were from 5 to 8 mm (Wu *et al.,* 2015). In order to determine the precise tetrad stage of each accession, the buds with lengths from 5 to 8 mm were harvested and divided into groups according to length, on a 0.5 mm scale. After petal removal, four anthers from each bud were selected, crushed and placed under the microscope. Only the anthers which contained typical tetrads were considered to be at tetrad stage. The anthers were harvested into tubes and stored at −70°C or immediately placed in liquid nitrogen for further analysis.

Extraction of RNA was carried out using a Spectrum Plant Total RNA Kit (cat. no. STRN250‐1KT; Sigma). In brief, *c*. 100 mg anthers were collected and ground in liquid nitrogen, and the subsequent steps were completed according to the manufacturer’s instructions. For the sequencing libraries, concentrated ploy‐A mRNA was fragmented and reverse‐transcribed using random primers. RNA samples were sent to Novogene (Tianjin, China) to construct standard Illumina transcriptome sequencing libraries.

### Reads mapping and variation calling

Low quality reads were eliminated using trimmomatic software (Bolger *et al.,* 2014). The remaining paired‐end reads were subsequently mapped to the allotetraploid cotton TM‐1 genome (Wang *et al.,* 2019) using the bwa software package, with default parameters (Li & Durbin, 2009). Sequencing reads produced by polymerase chain reaction (PCR) duplicates for each accession were filtered using picard, and only the unique mapped reads were retained in BAM format files. samtools and the Genome Analysis Toolkit (GATK) were used to perform SNP calling. In order to obtain high quality SNPs, the variations which were detected by both tools and covered by sequenced reads at least three times were retained for further analysis. Single nucleotide polymorphisms with a missing rate > 0.4 and a minor allele frequency (MAF) < 0.01 were discarded. Annotation of SNPs was performed using snpeff software (Cingolani *et al.,* 2012).

### Population genetic analysis

The SNPs with MAF > 0.05 were used for phylogenetic and population structure analysis. From this selection, randomly chosen SNPs were subjected to population structure analysis using the Structure software package (Falush *et al.,* 2003) with the following parameter: K2 to K9. We repeated this process at least five times. All compressed results files were then uploaded to online analysis toolkit structure harvester (http://taylor0.biology.ucla.edu/structureHarvester/) to perform consecutive analyses. A neighbor‐joining tree was constructed based on SNPs (MAF > 0.05) using phylip software (Lim & Zhang, 1999), and concomitantly visualized using the online tool itol (https://itol.embl.de/; Letunic & Bork, 2011). Principal component analysis (PCA) was performed using a combination of plink and gcat software. Single nucleotide polymorphisms with MAF > 0.05 were pooled in to plink software (‐‐make‐bed) to generate Gcat identifiable files (‐‐bfile ‐‐make‐grm) (Purcell *et al.,* 2007).

### Linkage disequilibrium

Linkage disequilibrium was calculated using tassel software with customized parameters (‐fork1 ‐ld ‐ldWinSize 1000000) (Bradbury *et al.,* 2007). The LD decay was calculated based on the values of *r* ^2^ between two SNPs and averaged in 1 kb windows with a maximum distance of 1000 kb. Linkage disequilibrium was calculated for each subgenome of allotetraploid cotton (At and Dt).

### Gene expression analysis and identification of long non‐coding RNAs

Quality controlled sequencing reads for each accession were mapped to the allotetraploid cotton reference TM‐1 genome using the hisat2 aligner (‐‐dta‐cufflinks ‐‐min‐intronlen 40 ‐‐max‐intronlen 100000) (Kim *et al.,* 2015a). Expression of protein coding genes (PCgenes) was predicted using cufflinks software (parameter ‐G) with an annotated ‘gff3’ file. Data normalization was performed by transforming mapped transcript reads to fragments per kilobase per million mapped fragments (FPKM). The software package rmats was used to identify intron retention (IR) events, and the genes which have IR in at least 20 accessions were harvested for analysis of overlapping SNPs and introns (Shen *et al.,* 2014).

While identifying long noncoding RNAs (lncRNAs), cufflinks analysis was carried out with parameter ‐g. With the ‘gff3’ file guiding identification, novel transcripts in each accession were merged by cuffmerge. cuffcompare processing was applied to compare assembled transcripts to the annotated genome information. After comparison, the transcript symbols (u: novel transcripts; x: antisense transcripts) in the ‘gtf’ file were extracted to identify lncRNAs as follows: (1) transcripts identified < 10 times were discarded (< 5% of 218); (2) transcripts originating from tRNA and rRNA were removed (blast E < 1 × 10^−5^); (3) transcripts showing protein coding potential against Swiss‐Prot and Pfam (blast E < 1 × 10^−5^) were eliminated; (4) transcripts that were deemed to have low coding potential by the coding‐non‐coding index (CNCI) tool were also discarded; and (5) transcripts with length < 200 bp were removed. Finally, all the sequencing reads were mapped to lncRNA transcripts to verify validity and expression levels.

### Genome‐wide association studies

Single nucleotide polymorphisms with MAF > 0.05 were obtained to perform association studies. To avoid false positives, population structure was regarded as a random effect by using the kinship (K) matrix in a mixed linear model (MLM). Single nucleotide polymorphisms with MAF > 0.05 were acquired for the calculation of K. By computing the genomic inflation factor (λ = 1.0020), false positive association signals arising as an effect of population structure could be controlled. We performed a GWAS using MLM embedded in the tassel software, and the GWAS threshold was set to −log_10_(1/*n*), according to the Bonferroni‐adjusted significance threshold. The genomic inflation factor was calculated using the *P*‐value of each SNP in the Matlab software package.

Association analysis in the LD block was performed using the ldheatmap package in R. The 280 kb LD decay rate was used to analyze predicted genes within the 560 kb interval in which the peak SNP was centered in the chromosome.

### Identification of expression quantitative trait loci (eQTLs)

Firstly, a quantile transformation was applied to normalize the expression level of each transcript using the ‘qqnorm’ function in R. The SNPs with MAF > 0.05 were used to assess the association between SNPs and normalized expression traits. The matrixeqtl software in R was used to identify positively associated SNPs (Shabalin, 2012). To increase the number of positive association results, the *P*‐value cutoff was set to *P* < 1 × 10^−6^ until a low *P*‐value reached 1 × 10^−20^. While identifying eQTLs, the significant associated SNPs with distances < 20 kb were classified into the same cluster, and a cluster with at least four significant SNPs was regarded as a putative eQTL. Consecutive eQTLs located within 20 kb were regarded as the same effect sites and were merged (Li *et al.,* 2013).

To identify the eQTL hotspots, the statistical algorithm described by Silva *et al*. (2014) was implemented. We applied different window sizes to scan the hotspots (1, 2, 5, 10, 20, 50 kb). Finally, 5 kb was set as the window size, as the average length of genomic sequences of genes was approximately 5 kb. Putative hotspots which affected > 5 genes and passed the false discovery rate (FDR) adjustment (0.05) were regarded as true hotspots.

### Co‐expression network construction and gene ontology analysis

The FPKM values after log transformation of PCgenes and lncRNAs of all accessions were integrated into one input file to construct a weighted gene co‐expression network using the wgcna package in R (Langfelder & Horvath, 2008). A median absolute deviation was calculated by using all log FPKM value. Genes which were expressed in > 10 accessions and whose absolute deviation ranged in 85% of median absolute deviation were obtained. The genes whose absolute deviation more than 85% of median absolute deviation were regarded as outliers and then were excluded. 73 989 genes were retained for construction of the network. Each module was set to analyze the correlation using the Pearson correlation coefficient, and gene ontology (GO) enrichment analysis was performed for enrichment in the test module compared to the reference group using Fisher’s exact test.

### Transcriptome‐wide association study

The fusion software package was used to perform the TWAS. *Cis*‐SNPs (located within 500 kb of the physical position of the gene) of PCgenes and lncRNAs were harvested as described in a previous study (Gusev *et al.,* 2016). Genes with no local eQTLs or *Cis*‐SNPs were removed to avoid potential false heritability estimation using gcat (embedded in fusion). To have the best expression prediction, the model choice (‐‐models top1, blup, bslmm, lasso, enet in fusion) was applied (top1, single best eQTL; blup, best linear unbiased predictor; bslmm, Bayesian sparse linear model; lasso, LASSO regression; enet, elastic‐net regression). After prediction, the genes with the best model performance (*R* ^2^ values of at least 0.1) were retained for further analysis (Wu *et al.,* 2018; Gusev *et al.,* 2019). Single nucleotide polymorphisms used in the GWAS were captured as reference SNPs for LD value, and *Z*‐scores were calculated using the ‘qnorm’ function in R, based on *P*‐value and effect size of SNPs from the GWAS.

### Tissue dissection

Tissue dissection was similar to the procedure we used previously (Y. Ma *et al.,* 2018). In brief, after HT stress treatment, anthers were fixed in 50% FAA (50 ml absolute ethanol, 10 ml 37% formaldehyde solution, 5 ml acetic acid and diluted with water to 100 ml) after removal of bracts and petals. Dehydration used a graded ethanol series (30%, 50%, 70%, 95% and 100%). Tissue was embedded in paraffin and sectioned at 10 μm thickness.

### Full‐length coding sequence cloning of *GhHRK1*

Two pairs of primers were designed to clone *GhHRK1* using overlap‐extension methods. Two fragments with respective sequence lengths of 1071 bp and 1832 bp were first cloned and then extended to amplify the full‐length sequence of *GhHRK1*. The related primers are listed in Table S2.

### Quantitative reverse‐transcription polymerase chain reaction (qRT‐PCR)

Using the M‐MLV (cat. no. M1701; Promega) system, 3 μg total RNA was reverse‐transcribed. An ABI 7500 Real Time PCR system was used to perform qRT‐PCR. The 2^−ΔΔCt^ method was used to calculate gene expression levels. The expression levels were normalized to internal control *GhUB7* (*Ghir_A11G011460*), to standardize RNA content and integrity.

### *In situ* hybridization

A 100 bp coding sequence fragment from *GhHRK1* was amplified using the qRT‐PCR primer (GhHRK1‐qRT‐F: 5′‐ CGATAAAGCATTGGGCGAC‐3′; GhHRK1‐qRT‐R: 5′‐ATGTTTGGTCTGTCTGAGGGG‐3′) and cloned into the pGEM‐T easy vector (cat. no. A137A; Promega). The hybridization procedure was performed as described previously (Min *et al.,* 2013). Briefly, antisense RNA was synthesized using the DIG RNA Labeling Kit (SP6/T7) (cat. no. 11175025910; Roche). Sections were dewaxed, rehydrated and treated using proteinase K (cat. no. 25530049; Invitrogen, dissolved in PBS to 1 μg ml^−1^) for 30 min at 37°C before pre‐hybridization. A quantity of 40–150 ng DIG‐labeled probe was used for each slide, and hybridized for 16–24 h at 42°C. Anti‐Digoxigenin‐AP (cat. no. 11093274910; Roche, dissolved in BSA solution at 1 : 2500) was used to immunize with Dig‐labeled RNA. Nitroblue tetrazolium (NBT)/5‐bromo‐4‐chloro‐3‐indolyl‐phosphate (BCIP) stock solution (cat. no. 1681451; Roche) was used to detect mRNA abundance on the section slide. The hybridization process was repeated twice.

### Arabidopsis growth conditions and temperature treatment

All Arabidopsis plants were grown in the growth chambers (PGC FLEX; Conviron, Winnipeg, Canada) under long‐day conditions with a 16 h : 8 h, light : dark photoperiod at 22°C. Mutants SALK_067606C (*hrk1‐1*) and SALK_129987C (*hrk1‐2*) were confirmed by genotyping PCR. While all accessions were undergoing the flowering period, > 15 plants of each accession were subjected to HT stress (31°C for 2 d) treatment and subsequently returned to normal temperature (NT) conditions. After 10–15 d recovery, the NT controlled and HT treated siliques were sampled and immediately soaked in 75% ethanol solution to decolorize overnight. A Nikon (Tokyo, Japan) SMZ‐25 stereo‐microscope was used to capture images of the seed setting in the siliques. The seed setting rate was calculated as the ratio of the number of setting seeds to the total number of ovules in a whole silique. Each assay was repeated twice with at least six siliques for each technical replicate.

For the Arabidopsis pollen staining, HT treated flowers at stage 13–14 were sampled, then immersed in TTC solution to stain for 30 min. The anthers were placed on glass slides and photographed under the microscope.

### *In vivo* and semi‐*in vivo* pollen tube elongation assays

For *in vivo* pollen tube elongation assays, flowers at floral stage 12 (1 d before anthesis) were emasculated for cross pollination. After pollination, the pistils were placed on solid pollen germination medium (Boavida & McCormick, 2007) for incubation in a growth chamber under NT and HT conditions for 20 h. Subsequently, the pistils were sampled and fixed in Carnoy’s Fluid (6 : 3 : 1, ethanol : chloroform : acetic acid) for at least 6 h. The fixed pistils were subsequently rehydrated using a graded ethanol series (75%, 50%, 30%, distilled water), followed by softening in 2 M NaOH overnight. For pollen tube staining, 0.1% (w/v) aniline blue solution (dissolved in 108 mM K_3_PO_4_, pH ~ 11) was applied. Images of whole pistils were captured using a Leica (Bensheim, Germany) SP8 Lightning confocal microscope to show the elongation of pollen tubes. Each assay was repeated for twice with at least 10 siliques for each technical replicate.

For semi‐*in vivo* pollen tube assay, styles of emasculated wild‐type (WT) pistils were cut and placed on the solid pollen germination medium for 4 h after pollination. The NT control was set at 22°C, while the HT treatment was set at 31°C in both semi‐*in vivo* and *in vivo* pollen tube germination assays.

### Transgenic complementation assays

The *GhHRK1* sequence fragment was cloned into the pMDC83 vector using the Gateway cloning system (cat. no. 11789013; Invitrogen). The constructed vector (*35S:GhHRK1‐GFP*) was introduced into WT, *hrk1‐1* and *hrk1‐2* using the floral dip method. A vector containing the *35S:GFP* fragment was simultaneously introduced into WT and mutants as a transgenic control.

## Results

### High temperature (HT) stress disrupts pollen activity differentially in different accessions

**Fig. 1:**
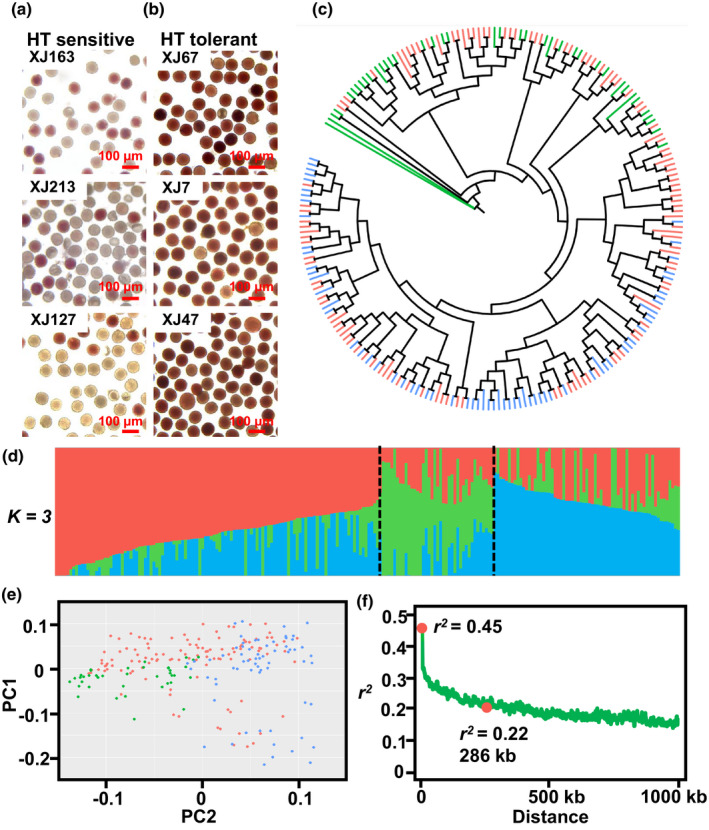
Phenotypic and genomic divergence analysis. (a, b) Typical images of 2,3,5‐Triphenyl tetrazolium chloride (TTC) staining of different cotton accessions under high temperature (HT) stress. Pollen grains with high viability (b) appear darker than those with low viability (a). XJ163, XJ213 and XJ127 are representative of HT‐sensitive accessions; XJ67, XJ7 and XJ47 are representative of HT‐tolerant accessions. (c) Neighbor‐joining tree for 218 upland cotton accessions, based on single nucleotide polymorphisms (SNPs) with a minor allele frequency (MAF) > 0.05. Branch colors indicate the different distributions of groups divided by population structure results. (d) Population structure based on *K* = 3. The left‐hand *y*‐axis quantifies genetic diversity in each accession (in red, green and blue), which is represented by a stack column. The subpopulation division is indicated by two dashed black lines. (e) Principal component analysis (PCA) of the first two principal components for all accessions. The accessions in the red group (indicated in (d)) show genetic similarity to accessions in both the green and blue groups. (f) Linkage disequilibrium (LD) decay rate analysis. The distance was estimated to be 286 kb (*r* ^2^ = 0.22) by calculating *r* ^2^ between two pairwise SNPs.

We performed phenotypic analysis of pollen viability among the 218 natural cotton accessions (Table S3). Where the accessions suffered high temperature (HT) stress (> 3 d on which the daily maximum temperature is > 37°C), the flowers were harvested for evaluation of pollen viability. 2,3,5‐Triphenyl tetrazolium chloride (TTC) staining and microscopic analysis were carried out to determine pollen viability for each cultivar (Fig. 1a,b). Under HT stress, 42 accessions showed very high pollen viability (i.e. > 90% of the pollen was viable) and 21 accessions had low viability (< 25%); the others exhibited intermediate pollen viability.

### RNA sequencing reveals genetic diversity among the accessions

Microspore sterility has been observed at the tapetum degradation stage following heat stress (Y. Ma *et al.,* 2018), the aberrant gene expressions and metabolism are expected to occur at an early developmental stage (Grienenberger *et al.,* 2010; Kim *et al.,* 2015b). In order to uncover the initial response to HT, we sampled anthers at the tetrad stage for each accession that suffered HT stress, and we performed RNA‐sequencing for both genotyping and transcriptome analysis (Tables S3, S4). After low‐quality‐read trimming and read mapping, a total of 3 352 750 SNPs were obtained. After filtering for missing rate < 0.4 and MAF > 0.01, 549 216 SNPs were identified for further analysis. The number of SNPs was much lower than that found for maize (Fu *et al.,* 2013) and lettuce (Zhang *et al.,* 2017) in a similar population set, suggesting there are fewer variations across the cotton accessions. To validate the SNPs, we compared SNPs from 38 accessions in this study with the variation data for these same cotton lines described in Wang *et al*. (2017). Our SNP data showed a 95.1% identity with the SNP data from Wang *et al*. (2017), suggesting a high degree of consistency between the datasets (Table S5). Analysis of the physical position of these SNPs revealed that a large number were positioned in intergenic and intron regions (Fig. S2a). The SNPs located in genic regions showed a relatively high abundance in coding regions rather than in the 3′‐UTR and 5′‐UTR (Fig. S2b,c). The IR events could lead to introns being retained in mRNA. Due to the high fraction of intron‐located SNPs, we analyzed IR events in the population (Table S6). We found that 54 293 genes had IR in at least 20 accessions, and 184 289 SNPs (33% percent of 549 216) were located in the retained introns. While only 2866 SNPs (0.5% percent of 549 216) were located in the spliced intron according to transcriptome data, the high IR frequency could explain the high fraction of intron located SNPs.

Following SNP annotation, the population genetic architecture was investigated. The SNPs were subjected to population structure analysis, phylogenetic tree construction, PCA and LD calculations. It was found that the 218 accessions could be organized into three genetic groups (Fig. S3), with 114 (red in Fig. 1c,d), 39 (green) and 65 (blue) samples in the three groups. The PCA showed that the accessions belonging to the green and blue groups were clustered separately, while the red group accessions were found in both clusters. The accessions belonging to the red group were all bred in China, while the samples classified into the green and blue groups were mainly derived from Russia, Kazakhstan and the USA (Figs 1e, S4). The LD decay physical distance was estimated at 286 kb, as pairwise *r^2^* from maximum (0.45) to half value (Figs 1f, S5). This value is slightly lower than the results reported by Wang *et al*. (2017) (296 kb) and Shen *et al*. (2019) (298 kb). Overall, our 218 accessions showed a natural LD and population structure, and were considered suitable for further analysis.

### Transcriptome data integration and co‐expression network analysis

Gene expression profiles under HT in anthers were investigated in our previous study using two different cotton lines (Min *et al.,* 2014; Y. Ma *et al.,* 2018), but the small sample size meant that the results were limited. In order to comprehensively understand the transcriptome divergence under HT stress in a large population, we first analyzed the expression of all transcripts, including protein coding genes (PCgenes) and long noncoding RNAs (lncRNAs), for each of the 218 accessions, and processed the samples by hierarchical clustering (Li *et al.,* 2016). In the clustering tree, accessions harboring parallel phenotypes were clustered into adjacent groups, relating transcriptome differences to phenotypic variations (Fig. S6).

**Fig. 2:**
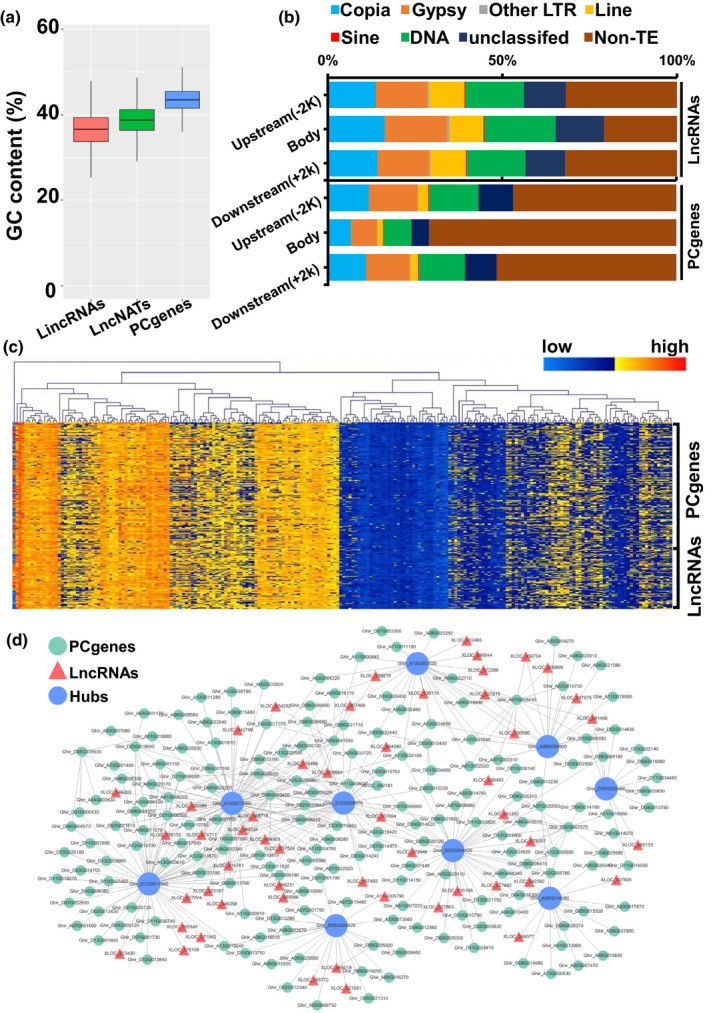
Integrated transcriptome analysis of cotton anther. (a) Guanine‐cytosine (GC) content analysis of long noncoding RNAs (lncRNAs) in cotton anther. Long intergenic RNAs (lincRNAs) and long noncoding natural antisense transcripts (lncNATs) both showed low GC content compared to protein coding genes (PCgenes). (b) Comparison of the transposable element (TE) overlap rate between lncRNAs and PCgenes. A large proportion of lncRNAs overlapped with TEs in the transcript body, but there were fewer TEs positioned in the PCgenes’ body. The icons represent the different kinds of TE shown in the stack plots. (c) Hierarchical clustering of genes in the ‘black’ module. The expression levels of PCgenes and lncRNAs showed similar patterns in clustered groups. 108 accessions displayed increased expression levels of 271 genes (shown on the left‐hand side, depicted in yellow), while 110 accessions showed decreased expression trends (right‐hand side, blue). (d) Co‐expression network of each gene in the ‘black’ module. The green circles represent PCgenes, and the red triangles denote lncRNAs. Nine hub PCgenes (shown as blue circles) were found to link all genes in the module.

LncRNAs perform pivotal functions in organ development and epigenetic modification of the genome in humans and other mammals (Iyer *et al.,* 2015). One lncRNA transcript generated from the antisense transcript of a heat shock factor gene (*HSFB2a*) was found to participate in gametophyte development in Arabidopsis (Wunderlich *et al.,* 2014). Comprehensive identification of lncRNAs during pollen development has been accomplished in *Brassica* (Huang *et al.,* 2018). However, there are few studies reporting the functions of lncRNAs in relation to heat stress in cotton anther development. The abundance of SNPs located in intergenic regions implied that a substantial proportion of the transcripts were possible lncRNAs (Fig. S2a). The RNA‐seq data was subsequently used to identify lncRNAs in all accessions. After merging loci and filtering for protein coding potential, a total of 26 158 lncRNA loci were identified (14 094 loci in the At subgenome and 12 064 loci in the Dt subgenome; Fig. 2a; Table S7). We overlapped the SNPs and the lncRNA loci, and found that *c*.16% (89 269 of 549 216) of the SNPs were located in the lncRNA regions. The lncRNAs in the cotton anther showed similar guanine‐cytosine (GC) content to those identified in cotton fiber and root (Wang *et al.,* 2015; Zhang *et al.,* 2018). In Arabidopsis, a large number of lncRNAs have been found to be derived from transposable elements (TEs), with unclear roles (Bohmdorfer *et al.,* 2016). We studied the composition of lncRNAs in TEs in tetraploid cotton anther, and found that lncRNAs overlapped much more with TEs than with PCgenes, especially in the lncRNA body region (Fig. 2b). Approximately 30% of lncRNA loci overlapped with long terminal repeats (LTRs) – the upland cotton genome comprises a large number of LTRs (Fig. S7). However, long interspersed nuclear elements (LINEs) and LTR/Copia were represented at relatively high proportions (15% and 20%) among the lncRNA loci, compared to their proportions across the whole genome (1.7% and 12% respectively). These results were similar to the observation that active lncRNAs are predominantly derived from LINEs in interspecific cotton hybrids (Zhao *et al.,* 2018). Interestingly, an unexpectedly high proportion of TEs overlapped with PCgenes’ upstream regions (the promoters) and downstream regions (almost 50% of PCgenes) (Fig. 2b). Transposable element transcription is usually suppressed by DNA methylation in order to stabilize the genome (Autran *et al.,* 2011), and the proportion of TEs overlapping with PCgenes suggested that gene expression in the cotton anther may be largely regulated via epigenetic mechanisms.

To understand the regulatory relationship between lncRNAs and PCgenes, we combined the expression data from PCgenes with lncRNA data, to construct a co‐expression network (soft thresholding power β = 28; cut‐off = 0.85) (Fig. S8). A total of 73 989 transcripts were divided into 16 modules (Fig. S9a), and genes in the designated ‘black’ module showed negative correlation with pollen viability (Pearson correlation, *r* = −0.16, *P* = 0.02 (Figs 2c, S9b–d). Expression profiles of genes in the other modules are displayed in Fig. S10.

The expression patterns of genes in the black module were investigated (Fig. 2c). 108 accessions with increased expression trends displayed lower pollen viability, indicating that these genes may perform negative regulatory functions in response to HT stress (Fig. S9d). The module includes 9 hub genes with 271 transcripts (209 PCgenes and 62 lncRNAs) which are involved in programmed cell death and hormone response (Fig. S11), indicating that a complex regulatory network is involved the HT stress response (Fig. 2d; Table S8).

### Genome‐wide association study identifies three loci associated with the HT stress response

**Fig. 3:**
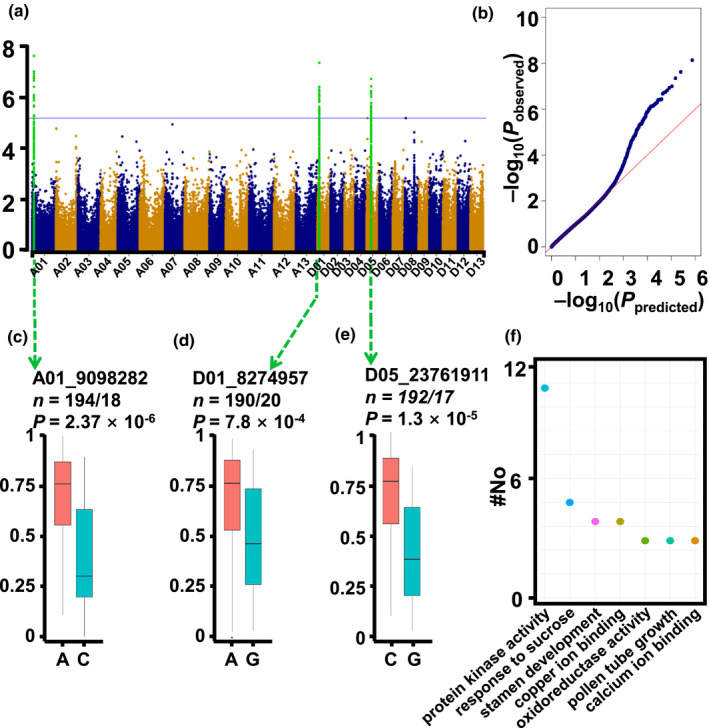
Association study for pollen activity under heat stress in cotton. (a) Manhattan plot for genome‐wide association study (GWAS). Single nucleotide polymorphisms (SNPs) located within the linkage disequilibrium (LD) decay distance are shown as light green dots. The horizontal line indicates the Bonferroni‐adjusted significance threshold (*P* = 6.3 × 10^−6^). (b) Quantile–quantile plot for the GWAS. In the mixed linear model (MLM), the false positive association could be controlled. (c–e) Pollen viability of corresponding haplotypes of peak SNPs in chromosomes A01, D01 and D05 among the population. The chromosome numbers and peak SNP positions are linked by the green arrows. Statistical significance was determined by a two‐tailed Student’s *t*‐test. Pollen viability for each haplotype is shown in the box plot. The *y*‐axis represents pollen viability. *n*, number of genotypes belonging to each haplotype group. (f) Number of candidate genes participating in specific pathways and functions located in associated intervals shown by GWAS.

Use of the GWAS technique has facilitated the identification of genes and QTLs linked to important agricultural traits (Si *et al.,* 2016; Wu *et al.,* 2019). In order to identify genes involved in the HT stress response in pollen grains, we collected 175 430 SNPs with MAF > 0.05 to perform a GWAS based on the pollen viability values. Association analysis conducted using these SNPs provided three loci associated with pollen viability at *P* < 6.3 × 10^−6^ (Bonferroni‐adjusted significance threshold 1/*n*, *n* = 175 430). The association results obtained using a mixed linear model are shown in the form of a Manhattan plot and a quantile–quantile plot in Fig. 3(a,b). The haplotypes based on the peak SNPs from three associated signals showed distinct association with the phenotype of pollen viability under HT stress (Fig. 3c–e). When comparing the three loci to the 160 QTLs of 16 traits reported previously (Huang *et al.,* 2017), we found there were no QTLs overlapping with these three loci, suggesting that the tolerance of HT in cotton may have an independent regulatory mechanism. The LD in upland cotton was estimated to be 280 kb, and the three associated loci contain 75 PCgenes (Fig. S12), of which 20 had no annotations (Table S9). Among the 55 annotated genes, 16 were annotated as potential protein kinases, with 10 genes homologous to the Arabidopsis gene *At4g27290*, encoding a G‐type lectin receptor kinase family protein. The other genes were annotated as being involved in the response to energy substances and stamen development (Fig. 3f).

### Transcriptome wide association study (TWAS) identified *Ghir_A01G006180*, a gene that confers male sterility under HT stress

Critical functions of genetic variants which influence gene expression, known as eQTLs, have been found to affect agronomic traits. Robust eQTL mapping has been carried out in maize (Fu *et al.,* 2013), rice (Wang *et al.,* 2010; Kuroha *et al.,* 2017), tomato (Ranjan *et al.,* 2016), lettuce (Zhang *et al.,* 2017) and cotton fiber (Li *et al.,* 2020). Based on the expression and SNP data, we carried out transcriptome‐wide eQTL identification. The SNPs with MAF > 0.05 were used to perform the association study, with the significance cutoff set at *P* < 1 × 10^−6^, until the lowest *P*‐value for the peak SNPs reached 1 × 10^−20^.

**Fig. 4:**
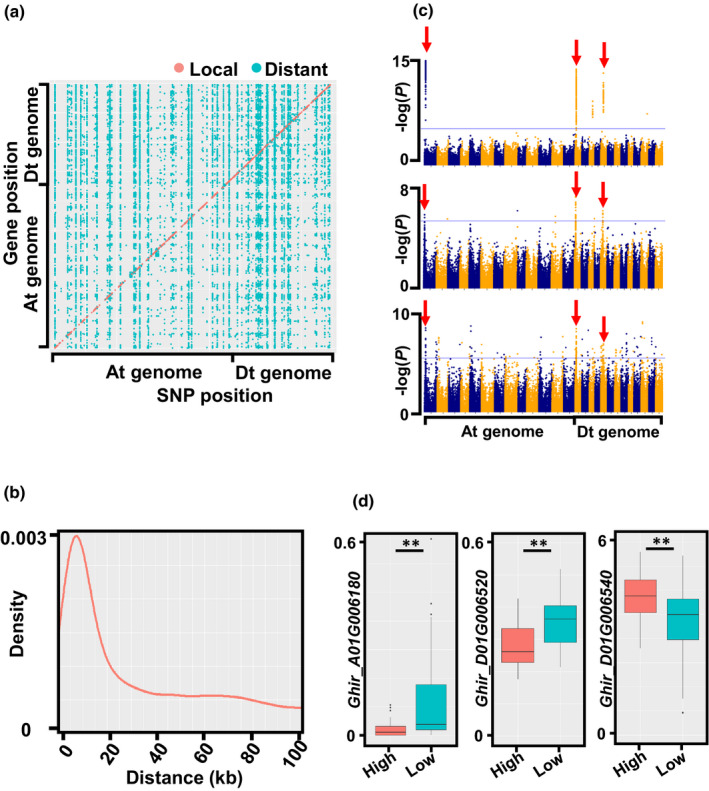
Expression quantitative trait locus (eQTL) analysis across the cotton genome. (a) Scatter plot showing the gene positions and associated single nucleotide polymorphisms (SNPs) with a Bonferroni‐adjusted significance threshold (*P* = 1 × 10^−6^). The SNPs which were located in local eQTLs were regarded as *Cis*‐SNPs (pink dots), while the other SNPs further away from the gene locations were classified as *Trans*‐SNPs (turquoise dots). (b) Genomic distance between eQTLs and affected genes across 100 kb. (c) Three Manhattan plots showing the intervals in chromosomes A01, D01 and D05 from a genome‐wide association study (GWAS; red arrows) as eQTLs. The Manhattan plots of *Ghir_A01G006180*, *Ghir_D01G006520* and *Ghir_D01G006540* are shown as examples. (d) Comparisons of the gene expression levels of *Ghir_A01G006180*, *Ghir_D01G006520* and *Ghir_D01G006540* among accessions with distinct pollen viability (shown as examples). The *y*‐axis represents the fragments per kilobase per million mapped fragments (FPKM) values of the corresponding genes. Asterisks indicate significant difference according to Student’s *t*‐test (**, *P* < 0.01).

A total of 32 396 eQTLs were identified, with effects on 8975 PCgenes and 4157 lncRNAs, covering 14% of the genes in the cotton genome (8975 of 70 198 PCgenes and 4157 of 26 158 lncRNAs) (Fig. 4a; Table S10). Among local eQTLs, the peak SNPs were mostly located within 10 kb of the affected genes (Fig. 4b). Individual eQTLs which could affect numerous (> 5) distant genes, known as eQTL hotspots, were also studied. We identified a total of 179 eQTL hotspots, which occupied 205 kb across the genome (Table S11). The intervals influencing pollen viability under heat stress on chromosomes A01, D01 and D05 were also found to be eQTL hotspots (Fig. 4c). The genes *Ghir_A01G006180*, *Ghir_D01G006520* and *Ghir_D01G006540*, which were candidate genes in the three QTLs, were also found to be influenced by eQTLs, indicating a solid association between gene expression and pollen viability in the cotton anther (Fig. 4c,d).

We compared our eQTL‐containing genes to the 125 genes contributing to fiber development that were published previously (Wang *et al.,* 2019) and found 19 overlapping genes, suggesting dissimilar regulatory mechanisms for fiber and male reproductive organs.

Based on the gene expression levels and *cis*‐SNP data, we further performed a TWAS to associate gene expressions with the different pollen viability phenotypes. According to our results (Fig. S13; Table S12), six genes (four PCgenes and two lncRNAs) showed significant association with the pollen viability trait, supporting the idea that disruption of gene expression at early developmental stages can result in male sterility under HT. Among these four PCgenes, three genes were represented in the 75 candidate PCgenes associated by the GWAS, the most significant being *Ghir_A01G006180*, suggesting that it is very likely that this gene is a component of the HT response in anthers.

### *GhHRK1* is a negative regulator during the HT stress response in early anther development

**Fig. 5:**
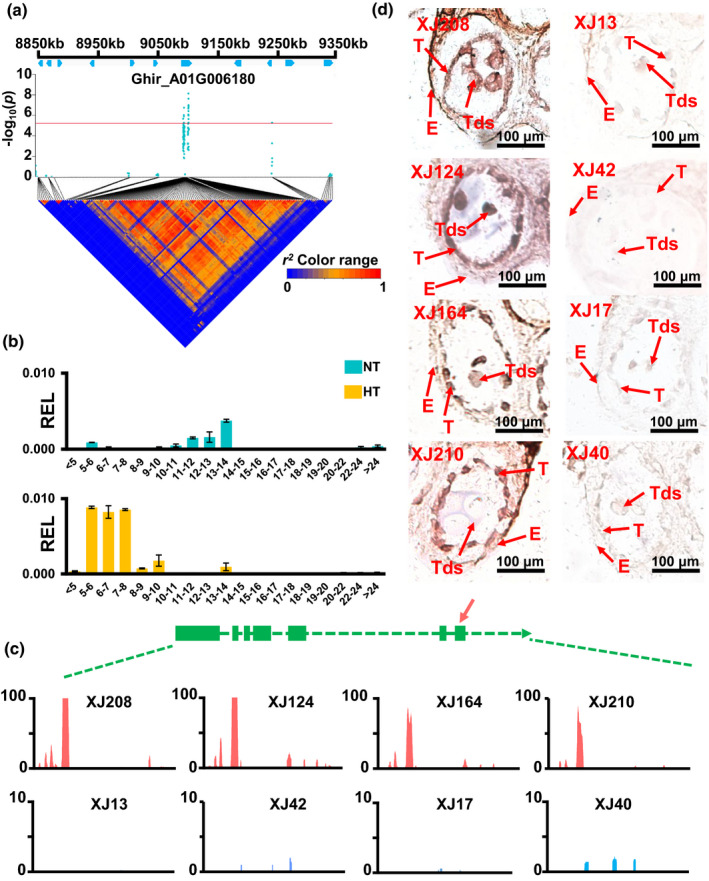
Functional characterization of *GhHRK1* in cotton anther. (a) Regional Manhattan plot and linkage disequilibrium (LD) analysis of the most significant interval in A01. *Ghir_A01G006180* was found to be associated with pollen viability in the LD analysis. (b) Expression profile analysis of *GhHRK1* in anthers of H05, a HT‐sensitive accession. In the early anther developmental stages of H05, expression of *GhHRK1* was increased under HT stress (shown as yellow columns). HT, high temperature; NT, normal temperature; R.E.L, relative expression level. The values are mean ± SD. (c) Gene structure analysis of *GhHRK1*. *GhHRK1* contained seven exons and showed a clear difference in mRNA abundance among different accessions under HT stress. The arrow indicates the fragment used for quantitative reverse‐transcription polymerase chain reaction (qRT‐PCR) and *in situ* hybridization. The *y*‐axis indicates coverage of sequencing reads for *GhHRK1*. (d) *In situ* hybridization of *GhHRK1* in anthers of different accessions under HT stress. *GhHRK1* was found to be expressed in tapetum and tetrads of the low viability accessions under HT stress. XJ208, XJ124, XJ162 and XJ210 are four HT‐sensitive accessions; XJ13, XJ42, XJ17 and XJ40 are four HT‐tolerant accessions. T, tapetum; Tds, tetrads; E, endothecium.

Thirteen significant SNPs (*P* < 6.3 × 10^−6^) located in *Ghir_A01G006180* showed an association with pollen viability (Fig. 5a, S14). According to the existing genome data (Wang *et al.,* 2019), the genomic sequence of *Ghir_A01G006180* comprises 13 887 bp, consisting of 14 exons with an open reading frame of 4785 bp. *Ghir_A01G006180* encodes a putative protein tyrosine kinase, with protein sequence similarity to the Arabidopsis G‐type lectin receptor kinase gene *At4g27290*. While analyzing the protein architecture of *Ghir_A01G006180*, we found that the protein domains seemed to be duplicated, while the homologous *At4g27290* only contained half of the protein domain structure of *Ghir_A01G006180* (Fig. S15). Likewise, only the first seven exons of *Ghir_A01G006180* were found to have read coverage in low pollen viability accessions (Fig. S16), which led us to question the existing gene model annotation data. We referred to another TM‐1 cotton genome dataset described by Hu *et al*. (2019), and found that the *Ghir_A01G006180* locus contained two genes, referred to as *GH_A01G*0682 and *GH_A01G0683*. After sequence alignment, we found there was an incorrect annotation at the *Ghir_A01G006180* locus – *GH*_*A01G0682* was the causal gene at the *Ghir_A01G006180* locus (Fig. S17). We finally designated *GH*_*A01G0682* as Heat‐related Receptor Kinase *GhHRK1*. The full‐length coding sequence of *GhHRK1* was cloned using overlap‐extension methods, and the sequence is given in Table S13. Using the refined gene model, we found that there were nine SNPs in the 3′ downstream of *GhHRK1*, one SNP located in the intron between the second and third exons, and three SNPs that could cause nonsynonymous mutations (Fig. S18; Table S14). The most notable difference in terms of *GhHRK1* between low viability and high viability accessions is the expression level. We therefore hypothesized that mis‐regulated expression of *GhHRK1* in an early developmental stage resulted in the male sterility in pollen grains. We first amplified a 2000 bp upstream fragment of *GhHRK1* from each accession to analyze the variations in the promoter. No significant structural variations were found among the accessions. We further annotated the regulatory motifs in the promoter using the plantcare online tool (Table S15). Aside from the conserved CAAT‐box and TATA‐box, there were four binding sites for MYB transcription factors, one motif for gibberellin response (GARE) and one motif for auxin response (AuxRR). In our previous study, *AtMYB24* transcription factor was upregulated in an early developmental stage of the anthers in Arabidopsis under HT stress (Li *et al.,* 2018). Meanwhile, an MYB transcription factor was found to be a negative regulator of the heat response in Arabidopsis seedlings, indicating that the unexpected expression of *GhHRK1* may be disordered by abnormal transcriptional regulation (Liao *et al.,* 2017). We further analyzed the expression of transcription factors related to MYB, GARE and AuxRR domains in the accessions showing distinct pollen viability. There were no notable differences in expression levels between the GARE and AuxRR transcription factors. However, some MYB transcription factors showed higher expression levels in the low pollen viability accessions, which may explain the disordered expression of *GhHRK1* at an early stage (Fig. S19).

To test the hypothesis that *GhHRK1* contributes to the heat stress response in cotton, we first analyzed the expression of *GhHRK1* in our HT‐sensitive cotton genotype H05. We found that *GhHRK1* was expressed in early meiosis (5–6 mm buds), at the tapetum degradation stage (9–14 mm buds) and at the anther dehiscent stage (> 24 mm buds) under NT conditions. High temperature stress disrupted the expression profile of *GhHRK1*, leading to an increased expression level at early developmental stages (5–10 mm buds) but a decreased level at the tapetum degradation stage (Fig. 5b). We determined the variations in expression of *GhHRK1* across the population. The accessions which exhibited low pollen viability under HT stress showed relatively high accumulations of *GhHRK1* mRNA transcripts (Fig. 5c). *In situ* hybridization analysis was carried out in eight accessions which exhibited distinct pollen viability under HT conditions (Fig. S20). Samples subjected to HT stress showed that *GhHRK1* was abundant in the tapetum and tetrads in the low viability accessions at tetrad stage, suggesting that *GhHRK1* expression was associated with a loss of pollen viability in response to HT stress in the early cotton anther, and may be part of a negative regulatory pathway of cell viability (Fig. 5d). We also investigated the expression of *GhHRK1* at the tapetum degradation stage in the same accessions. Increased expression of *GhHRK1* was found in the microspores and tapetum of high pollen viability accessions, while low expression of *GhHRK1* and aberrant microspore structure were present in the low viability accessions. This suggests that *GhHRK1* may normally be activated at the tapetum degradation stage (Fig. S21).

### Arabidopsis homologous *GhHRK1* mutant displayed enhanced HT tolerance

**Fig. 6:**
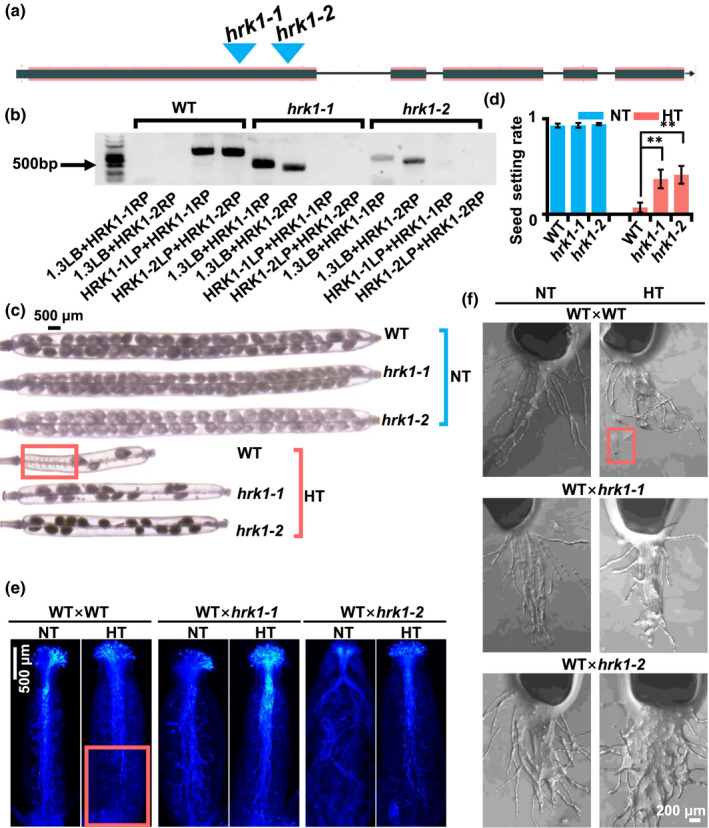
The Arabidopsis homologous *GhHRK1* mutant showed enhanced HT stress tolerance. (a) Gene model of Arabidopsis homologous gene of *GhHRK1* (referred as *At4g27290* in the main text). Blue triangles indicate the T‐DNA insertion positions according to The Arabidopsis Information Resource (TAIR) website. *hrk1‐1,* SALK_067606C; *hrk1‐2,* SALK_129987C. (b) Mutant genotyping using polymerase chain reaction (PCR). Our findings confirmed that *hrk1‐1* and *hrk1‐2* carried T‐DNA in the target gene region. (c) Images of decolorized siliques of wild‐type (WT) and mutants under normal temperature (NT) control conditions and high temperature (HT) treatment. The number of seeds decreased more in the WT than in the mutants (indicated by a red rectangle) under HT treatment, while there were no significant differences in seed setting rates among the three lines under NT conditions. (d) A histogram plot for seed setting rates of WT and mutants under NT and HT conditions, respectively. Asterisks indicate significant difference according to Student’s *t*‐test (**, *P* < 0.01). The values are mean ± SD. (e) *In vivo* pollen tube elongation assays of WT, *hrk1‐1* and *hrk1‐2*. Both WT and mutant pollen tubes reached the bottom of the pistils under NT conditions, while HT stress notably repressed the pollen elongation in WT (indicated by a red rectangle). (f) Semi‐*in vivo* pollen tube elongation assays of WT, *hrk1‐1* and *hrk1‐2*. Pollen tubes of *hrk1‐1* and *hrk1‐2* elongated normally under HT stress, while WT pollen tubes elongated less than those in mutants. Exposure of pollen tubes could be was observed in WT pollen tubes under HT stress (indicated by a red rectangle).

According to The Arabidopsis Information Resource website (TAIR, https://www.arabidopsis.org/), there are 23 G‐type lectin receptor kinase genes in Arabidopsis. The homologous analysis and phylogenetic tree showed that *At4g27290* is most closely adjacent to *GhHRK1* (Fig. S22). We next searched Arabidopsis mutant pools to find the homologues of *GhHRK1* (referred to as *At4g27290*) to elucidate the biological function of *At4g27290* under HT stress in Arabidopsis. Two mutant lines, SALK_067606C (*hrk1‐1*) and SALK_129987C (*hrk1‐2*) were obtained from the SALK Confirmed T‐DNA Project (Alonso *et al.,* 2003). According to data from the gene model (Fig. 6a) and the flanking sequence on the TAIR website, two mutant lines were confirmed to be carrying target T‐DNA (Fig. 6b). These two mutant lines showed reinforced vegetative development under NT conditions during the seedling period (Fig. S23). Higher pollen viability was also found in the mutant pollen grains after 3 d of HT treatment (Fig. S24). Few results have been reported regarding the function of *At4g27290*, but it has been shown to have potential functions relating to cell polarity (Bruex *et al.,* 2012), implying that this gene may function in the pollen tube elongation process during HT stress. Therefore, the WT Col‐0 line and mutant lines *hrk1‐1* and *hrk1‐2* were subjected to HT treatment (31°C for 2 d) at the flowering stage. A higher seed setting rate was found in the mutants after HT treatment, compared to WT, while there was no obvious difference in seed setting rate between the WT and mutants under NT conditions (Figs 6c,d, S25). Interestingly, no seeds were found at the bottom part of any of the examined siliques from the WT plants under HT conditions, indicating that almost no pollen tubes had reached the bottoms of the siliques (Fig. 6c,d).

To validate this inference, an *in vivo* pollen tube elongation assay was carried out under NT and HT conditions, in both WT and mutants. Three pollen tube elongation assays were conducted, referred as WT × WT, WT × *hrk1‐1* and WT × *hrk1‐2*. The emasculated WT pistils were pollinated and subsequently subjected to a HT treatment, in which they were placed in chamber at 31°C for 20 h, while the pistils under 22°C were set as control. Pollen tubes of all three combinations reached the bottom of the pistils under NT conditions. Under HT conditions however, WT pollen tubes only reached the mid‐point of the pistils. The pollen tubes of *hrk1‐1* and *hrk1‐2* elongated to the bottom of the pistils, as normal, under HT stress (Fig. 6e). To dissect the phenotype of shorter pollen tubes of WT under HT stress, a semi‐*in vivo* pollen tube assay was carried out as per the *in vivo* pollen tube assay. The pollen tubes elongated normally for all three combinations under NT control conditions; however, HT stress repressed the elongation of WT pollen tubes and resulted in the exposure of pollen tubes in WT (Fig. 6f). These results indicate that *At4g27290* can clearly affect pollen viability, explaining why the WT pollen tubes could not reach the bottom of the pistils and why there were no setting seeds in the bottom parts of the siliques.

**Fig. 7:**
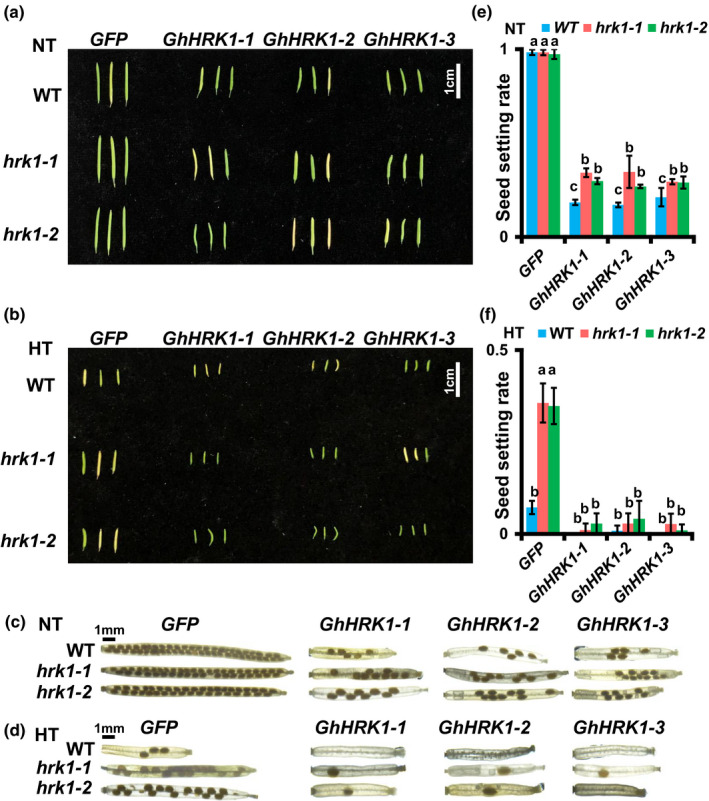
Phenotypic analysis of *GhHRK1* transgenic Arabidopsis individuals. (a) Overview of siliques of *GhHRK1* transgenic individuals under normal temperature (NT) conditions. Wild‐type (WT) and mutants containing *GhHRK1* showed decreased silique length. (b) Overview of siliques of *GhHRK1* transgenic individuals under high temperature (HT) stress. Wild‐type and mutants containing *GhHRK1* showed similar silique lengths, while mutants expressing *GFP* had a longer silique length than WT. (c) Images of seed setting in siliques under NT conditions. Wild‐type and mutants expressing *GFP* had similar seed setting. Introduction of *GhHRK1* reduced seed setting in both WT and mutants. (d) Images of seed setting in siliques under HT conditions. Wild‐type and mutants expressing *GhHRK1* had almost no seeds in their siliques. (e) Measurement of the seed setting rate of transgenic individuals under NT conditions. Values not sharing a common lowercase letter are considered significantly different (shortest significant range; *P* < 0.05). The values are mean ± SD. (f) Measurement of seed setting rate of transgenic individuals under HT stress. Values not sharing a common lowercase letter are considered significantly different (shortest significant range; *P* < 0.05). The values are mean ± SD.

To confirm the functional similarity of *GhHRK1* and *At4g27290*, we introduced *GhHRK1* into WT, *hrk1‐1* and *hrk1‐2* (Fig. S26). The transformation of *35S::GFP* was used as a control. Under NT conditions lower pollen viability and seed setting rate were detected, both in transgenic complement WT and mutant lines (Figs 7a,c,e, S27). Meanwhile, we found much more severe male sterility and a markedly decreased seed setting rate, simultaneously, in *GhHRK1* transgenic complement lines under HT stress (Figs 7b,d,f, S27). These results suggest that *GhHRK1* may have a similar function to *At4g27290*, playing a critical role in the HT response in plants.

## Discussion

Anther development is a genetically complex process (Zhao *et al.,* 2016) that can be adversely influenced by environmental stresses (Stromme *et al.,* 2015). The results described in this study show that RNA‐sequencing can be used to associate important QTLs involved in male reproductive development with a stress response in a complex crop genome. The transcriptome‐wide association study technique was also shown to be more efficient for the positioning of transcriptional regulators of male reproduction than the use of less rigorous correlation analyses (such as the Pearson correlation coefficient). Genes that respond to heat stress have been characterized in Arabidopsis, rice and tomato (Li *et al.,* 2015; Shen *et al.,* 2015), but the link between stress and male reproductive development is much less well understood. Transcriptome divergence could explain the molecular basis of phenotypic differences, via comparisons of the functions of differentially expressed genes. In our previous study, the altered auxin and sugar metabolism pathway was found to influence male fertility under HT stress through a comparison between two samples (Min *et al.,* 2014). However, it is difficult to understand comprehensively the mechanisms underlying a phenotype using such limited data. The current study benefited from the availability of a large number of accessions. By constructing a co‐expression network, several lncRNAs and PCgenes were found to participate in the response to HT stress. This revealed for the first time a role for lncRNAs in male reproduction under HT (Table S7).

A GWAS provides association analysis, and eQTL maps have achieved single‐gene resolution cloning in rice (Si *et al.,* 2016) and maize (Li *et al.,* 2019). In our study, three loci were found by GWAS analysis to respond to HT, showing that GWASs can be used to dissect the genetic regulation of traits in allopolyploid cotton as well as in diploid plant species. To connect trait and candidate gene expression, some researchers directly test preliminary correlations (such as Pearson’s correlation) to construct associations in the panels in which traits and expressions both exist, and this has generated reliable results (Wainberg *et al.,* 2019). However, analyses for which no RNA‐seq was available, but which relied on genotyping and phenotyping, were inefficient at identifying regulators because of their limited expression data. The TWAS technique aims to solve this problem and is becoming a powerful tool which provides abundant expression and association data aided by improved algorithms. The fact that TWASs have been successfully implemented in cotton fiber (Li *et al.,* 2020) and rapeseed (Tang *et al.,* 2020) provided motivation to conduct a TWAS in cotton anther. By applying TWAS, *GhHRK1* was associated with a pollen phenotype, and it is one of 75 PCgenes detected by the GWAS, suggesting that TWASs can be used to support the findings of GWAS analyses and so facilitate the identification of causal factors more precisely.

Mis‐expression of *GhHRK1* at the tetrad stage has been shown to result in male sterility in HT‐sensitive cotton accessions, suggesting that *GhHRK1* acts as a negative regulator in the HT stress response. It has also been demonstrated that 3 d HT treatment can result in pollen sterility at the anther dehiscent stage in WT Arabidopsis, indicating that *At4g27290* performs similar functions in the HT stress response at the early anther development stage. Introduction of *GhHRK1* into Arabidopsis mutants results in male sterility under HT conditions, possibly indicating that the mechanism of the HT stress response is conserved across the plant kingdom. In summary, we used RNA‐sequencing to construct the first transcriptome‐wide variation maps for the cotton anther, and linked genetic variation to HT tolerance by phenotyping pollen viability. These results provide potential targets for the improvement of cotton to satisfy the demand of high yield, multi‐resistant breeding.

## Author contributions

XZ and XL conceived and designed the project. XZ and XL collected accessions and designed the field arrangements. JW, Yajun Liang, and ZG performed field management and sampling. LM, Yaoyao Li, YW, YD, MW and QH performed anther sampling and RNA extraction. SX, CW, YZ, QF, Yanlong Li, HC, MC, AHK, XS and YM performed pollen staining and pollen counts. YM and MW analyzed the data. YM drafted the manuscript, and XZ, KL and LZ revised it. YM, LM and JW contributed equally to this work.

## Supporting information


**Fig. S1** Images of the experimental fields in Alear, Wuhan, Turpan and the glasshouse in Wuhan.
**Fig. S2** Analysis of the positions and potential functions of single nucleotide polymorphisms (SNPs).
**Fig. S3** Analysis of Evanno’s △*K* from the structure results.
**Fig. S4** Detailed information of the principal component analysis (PCA) of three subpopulations.
**Fig. S5** Linkage disequilibrium decay rate in At and Dt subgenomes.
**Fig. S6** Hierarchical clustering of all accessions.
**Fig. S7** Proportions of different kinds of transposable element (TE) in the genome.
**Fig. S8** Analysis of network topology using different soft‐thresholding powers.
**Fig. S9** Module classification and correlation analysis of each module.
**Fig. S10** Heatmap of expression profile of 15 modules.
**Fig. S11** Gene ontology (GO) analysis of genes in the ‘black’ module.
**Fig. S12** Expression levels of putative associated genes in different genotype accessions.
**Fig. S13** Transcriptome‐wide association study based on expression imputation with *cis*‐SNPs.
**Fig. S14** Association analysis in significant intervals in D01 and D05 chromosomes.
**Fig. S15** Protein domain analysis of *Ghir_A01G006180*, *At4g27290* and *GH_A01G0682*.
**Fig. S16** The coverage of sequencing reads for *Ghir_A01G006180* in eight accessions with different phenotypes.
**Fig. S17** Nucleic acid sequence alignment of *Ghir_A01G006180*, *GH_A01G0682* and *GH_A01G0683*.
**Fig. S18** Functional annotation of 13 significantly associated SNPs in *GhHRK1*.
**Fig. S19** Differentially expressed MYB transcription factors in accessions with distinct phenotypes.
**Fig. S20** Pollen viability images of accessions selected for *in situ* hybridization.
**Fig. S21** *In situ* hybridization of *GhHRK1* at the tapetum degradation stage for the same accessions as in Fig. 5.
**Fig. S22** Phylogenic analysis of *GhHRK1* and transcripts of G‐type lectin protein kinase in Arabidopsis.
**Fig. S23** An overview image of vegetative development of the wild‐type (WT) and two *hrk1* mutant lines during the seedling period.
**Fig. S24** Pollen viability of the WT and two *hrk1* mutants under normal temperature (NT) control and high‐temperature (HT) stress conditions.
**Fig. S25** Images of siliques and inflorescence of WT, *hrk1‐1* and *hrk1‐2* after HT treatment.
**Fig. S26** Transgenic complementation assay of *GhHRK1* in Arabidopsis.
**Fig. S27** Phenotypic analysis of *GhHRK1* transgenic individuals under NT, and HT conditions.

**Table S1** Meteorological data for Alear, Turpan and Wuhan.
**Table S2** Primers used in this study.
**Table S3** Summary of accession IDs and phenotypes of pollen viability.
**Table S4** Summary of RNA sequencing reads data used in this study.
**Table S5** Summary of SNP validation.
**Table S6** Summary of intron retained genes.
**Table S7** Summary of long noncoding RNA data.
**Table S8** Gene interaction information in the ‘black’ module.
**Table S9** Annotation information for 75 candidate protein coding genes.
**Table S10** Summary of eQTLs in the genome.
**Table S11** Detailed information of eQTL hotspots.
**Table S12** Transcripts associated with pollen viability identified by transcriptome‐wide association study.
**Table S13** Full‐length coding sequence for *GhHRK1*.
**Table S14** Annotation of significantly associated SNPs in *GhHRK1*.
**Table S15** Annotation of motifs in the promoter of *GhHRK1*.
Please note: Wiley Blackwell are not responsible for the content or functionality of any Supporting Information supplied by the authors. Any queries (other than missing material) should be directed to the *New Phytologist* Central Office.

## Data Availability

All of the data generated in the current study are available from the NCBI Sequence Read Archive (SRA) under accession no. PRJNA393079.

## References

[nph17325-bib-0001] Abbas M , Hernandez‐Garcia J , Blanco‐Tourinan N , Aliaga N , Minguet EG , Alabadi D , Blazquez MA . 2018. Reduction of indole‐3‐acetic acid methyltransferase activity compensates for high‐temperature male sterility in *Arabidopsis* . Plant Biotechnology Journal 16: 272–279. 28574629 10.1111/pbi.12768 PMC5785359

[nph17325-bib-0002] Alonso JM , Stepanova AN , Leisse TJ , Kim CJ , Chen H , Shinn P , Stevenson DK , Zimmerman J , Barajas P , Cheuk R *et al*. 2003. Genome‐wide insertional mutagenesis of *Arabidopsis thaliana* . Science 301: 653–657. 12893945 10.1126/science.1086391

[nph17325-bib-0003] Autran D , Baroux C , Raissig MT , Lenormand T , Wittig M , Grob S , Steimer A , Barann M , Klostermeier UC , Leblanc O *et al*. 2011. Maternal epigenetic pathways control parental contributions to *Arabidopsis* early embryogenesis. Cell 145: 707–719. 21620136 10.1016/j.cell.2011.04.014

[nph17325-bib-0004] Begcy K , Nosenko T , Zhou L‐Z , Fragner L , Weckwerth W , Dresselhaus T . 2019. Male sterility in maize after transient heat stress during the tetrad stage of pollen development. Plant Physiology 181: 683–700. 31378720 10.1104/pp.19.00707 PMC6776839

[nph17325-bib-0005] Boavida LC , McCormick S . 2007. Temperature as a determinant factor for increased and reproducible in vitro pollen germination in *Arabidopsis thaliana* . The Plant Journal 52: 570–582. 17764500 10.1111/j.1365-313X.2007.03248.x

[nph17325-bib-0006] Bohmdorfer G , Sethuraman S , Rowley MJ , Krzyszton M , Rothi MH , Bouzit L , Wierzbicki AT . 2016. Long non‐coding RNA produced by RNA polymerase V determines boundaries of heterochromatin. eLife 5: 19092. 10.7554/eLife.19092 PMC5079748 27779094

[nph17325-bib-0007] Bolger AM , Lohse M , Usadel B . 2014. Trimmomatic: a flexible trimmer for Illumina sequence data. Bioinformatics 30: 2114–2120. 24695404 10.1093/bioinformatics/btu170 PMC4103590

[nph17325-bib-0008] Bradbury PJ , Zhang Z , Kroon DE , Casstevens TM , Ramdoss Y , Buckler ES . 2007. TASSEL: software for association mapping of complex traits in diverse samples. Bioinformatics 23: 2633–2635. 17586829 10.1093/bioinformatics/btm308

[nph17325-bib-0009] Bruex A , Kainkaryam RM , Wieckowski Y , Kang YH , Bernhardt C , Xia Y , Zheng X , Wang JY , Lee MM , Benfey P *et al*. 2012. A gene regulatory network for root epidermis cell differentiation in *Arabidopsis* . PLoS Genetics 8: e1002446. 22253603 10.1371/journal.pgen.1002446 PMC3257299

[nph17325-bib-0010] Chen K , Guo T , Li XM , Zhang YM , Yang YB , Ye WW , Dong NQ , Shi CL , Kan Y , Xiang YH *et al*. 2019. Translational regulation of plant response to high temperature by a dual‐function tRNA^His^ guanylyltransferase in rice. Molecular Plant 12: 1123–1142. 31075443 10.1016/j.molp.2019.04.012

[nph17325-bib-0011] Cingolani P , Platts A , le Wang L , Coon M , Nguyen T , Wang L , Land SJ , Lu X , Ruden DM . 2012. A program for annotating and predicting the effects of single nucleotide polymorphisms, SnpEff: SNPs in the genome of *Drosophila melanogaster* strain w1118; iso‐2; iso‐3. Fly 6: 80–92. 22728672 10.4161/fly.19695 PMC3679285

[nph17325-bib-0012] Dai JL , Kong XQ , Zhang DM , Li WJ , Dong HZ . 2017. Technologies and theoretical basis of light and simplified cotton cultivation in China. Field Crops Research 214: 142–148.

[nph17325-bib-0013] Ding Y , Ma Y , Liu N , Xu J , Hu Q , Li Y , Wu Y , Xie S , Zhu L , Min L *et al*. 2017. microRNAs involved in auxin signalling modulate male sterility under high‐temperature stress in cotton (*Gossypium hirsutum*). The Plant Journal 91: 977–994. 28635129 10.1111/tpj.13620

[nph17325-bib-0014] Falush D , Stephens M , Pritchard JK . 2003. Inference of population structure using multilocus genotype data: linked loci and correlated allele frequencies. Genetics 164: 1567–1587. 12930761 10.1093/genetics/164.4.1567 PMC1462648

[nph17325-bib-0015] Fu J , Cheng Y , Linghu J , Yang X , Kang L , Zhang Z , Zhang J , He C , Du X , Peng Z *et al*. 2013. RNA sequencing reveals the complex regulatory network in the maize kernel. Nature Communications 4: 2832. 10.1038/ncomms3832 24343161

[nph17325-bib-0016] Grienenberger E , Kim SS , Lallemand B , Geoffroy P , Heintz D , Souza Cde A , Heitz T , Douglas CJ , Legrand M . 2010. Analysis of *TETRAKETIDE alpha‐PYRONE REDUCTASE* function in *Arabidopsis thaliana* reveals a previously unknown, but conserved, biochemical pathway in sporopollenin monomer biosynthesis. Plant Cell 22: 4067–4083. 21193572 10.1105/tpc.110.080036 PMC3027178

[nph17325-bib-0017] Gusev A , Ko A , Shi H , Bhatia G , Chung W , Penninx BW , Jansen R , de Geus EJ , Boomsma DI , Wright FA *et al*. 2016. Integrative approaches for large‐scale transcriptome‐wide association studies. Nature Genetics 48: 245–252. 26854917 10.1038/ng.3506 PMC4767558

[nph17325-bib-0018] Gusev A , Lawrenson K , Lin X , Lyra PC Jr , Kar S , Vavra KC , Segato F , Fonseca MAS , Lee JM , Pejovic T *et al*. 2019. A transcriptome‐wide association study of high‐grade serous epithelial ovarian cancer identifies new susceptibility genes and splice variants. Nature Genetics 51: 815–823. 31043753 10.1038/s41588-019-0395-x PMC6548545

[nph17325-bib-0019] Hu Y , Chen J , Fang L , Zhang Z , Ma W , Niu Y , Ju L , Deng J , Zhao T , Lian J *et al*. 2019. *Gossypium barbadense* and *Gossypium hirsutum* genomes provide insights into the origin and evolution of allotetraploid cotton. Nature Genetics 51: 739–748. 30886425 10.1038/s41588-019-0371-5

[nph17325-bib-0020] Huang C , Nie X , Shen C , You C , Li W , Zhao W , Zhang X , Lin Z . 2017. Population structure and genetic basis of the agronomic traits of upland cotton in China revealed by a genome‐wide association study using high‐density SNPs. Plant Biotechnology Journal 15: 1374–1386. 28301713 10.1111/pbi.12722 PMC5633765

[nph17325-bib-0021] Huang L , Dong H , Zhou D , Li M , Liu Y , Zhang F , Feng Y , Yu D , Lin S , Cao J . 2018. Systematic identification of long non‐coding RNAs during pollen development and fertilization in *Brassica rapa* . The Plant Journal 96: 203–222. 29975432 10.1111/tpj.14016

[nph17325-bib-0022] Iyer MK , Niknafs YS , Malik R , Singhal U , Sahu A , Hosono Y , Barrette TR , Prensner JR , Evans JR , Zhao S *et al*. 2015. The landscape of long noncoding RNAs in the human transcriptome. Nature Genetics 47: 199–208. 25599403 10.1038/ng.3192 PMC4417758

[nph17325-bib-0023] Khan AH , Min L , Ma Y , Wu Y , Ding Y , Li Y , Xie S , Ullah A , Shaban M , Manghwar H *et al*. 2020. High day and night temperatures distinctively disrupt fatty acid and jasmonic acid metabolism, inducing male sterility in cotton. Journal of Experimental Botany 71: 6128–6141. 32640017 10.1093/jxb/eraa319

[nph17325-bib-0024] Kim D , Landmead B , Salzberg SL . 2015a. HISAT: a fast spliced aligner with low memory requirements. Nature Methods 12: 357–U121. 25751142 10.1038/nmeth.3317 PMC4655817

[nph17325-bib-0025] Kim MJ , Kim M , Lee MR , Park SK , Kim J . 2015b. *LATERAL ORGAN BOUNDARIES DOMAIN* (*LBD*)*10* interacts with *SIDECAR POLLEN/LBD27* to control pollen development in *Arabidopsis* . The Plant Journal 81: 794–809. 25611322 10.1111/tpj.12767

[nph17325-bib-0026] Kuroha T , Nagai K , Kurokawa Y , Nagamura Y , Kusano M , Yasui H , Ashikari M , Fukushima A . 2017. eQTLs regulating transcript variations associated with rapid internode elongation in deepwater rice. Frontiers in Plant Science 8: 1753. 29081784 10.3389/fpls.2017.01753 PMC5645499

[nph17325-bib-0027] Langfelder P , Horvath S . 2008. WGCNA: an R package for weighted correlation network analysis. BMC Bioinformatics 9: 559. 19114008 10.1186/1471-2105-9-559 PMC2631488

[nph17325-bib-0028] Letunic I , Bork P . 2011. Interactive Tree Of Life v2: online annotation and display of phylogenetic trees made easy. Nucleic Acids Research 39: W475–W478. 21470960 10.1093/nar/gkr201 PMC3125724

[nph17325-bib-0029] Li H , Durbin R . 2009. Fast and accurate short read alignment with Burrows‐Wheeler transform. Bioinformatics 25: 1754–1760. 19451168 10.1093/bioinformatics/btp324 PMC2705234

[nph17325-bib-0030] Li H , Peng Z , Yang X , Wang W , Fu J , Wang J , Han Y , Chai Y , Guo T , Yang N *et al*. 2013. Genome‐wide association study dissects the genetic architecture of oil biosynthesis in maize kernels. Nature Genetics 45: 43–50. 23242369 10.1038/ng.2484

[nph17325-bib-0031] Li J , Zhu L , Hull JJ , Liang S , Daniell H , Jin S , Zhang X . 2016. Transcriptome analysis reveals a comprehensive insect resistance response mechanism in cotton to infestation by the phloem feeding insect *Bemisia tabaci* (whitefly). Plant Biotechnology Journal 14: 1956–1975. 26923339 10.1111/pbi.12554 PMC5042180

[nph17325-bib-0032] Li N , Lin B , Wang H , Li X , Yang F , Ding X , Yan J , Chu Z . 2019. Natural variation in *ZmFBL41* confers banded leaf and sheath blight resistance in maize. Nature Genetics 51: 1540–1548. 31570888 10.1038/s41588-019-0503-y

[nph17325-bib-0033] Li XM , Chao DY , Wu Y , Huang X , Chen K , Cui LG , Su L , Ye WW , Chen H , Chen HC *et al*. 2015. Natural alleles of a proteasome alpha2 subunit gene contribute to thermotolerance and adaptation of African rice. Nature Genetics 47: 827–833. 25985140 10.1038/ng.3305

[nph17325-bib-0034] Li Y , Min L , Zhang L , Hu Q , Wu Y , Li J , Xie S , Ma Y , Zhang X , Zhu L . 2018. Promoters of *Arabidopsis Casein kinase I‐like 2* and *7* confer specific high‐temperature response in anther. Plant Molecular Biology 98: 33–49. 30145767 10.1007/s11103-018-0760-7

[nph17325-bib-0035] Li Z , Wang P , You C , Yu J , Zhang X , Yan F , Ye Z , Shen C , Li B , Guo K *et al*. 2020. Combined GWAS and eQTL analysis uncovers a genetic regulatory network orchestrating the initiation of secondary cell wall development in cotton. New Phytologist 226: 1738–1752. 10.1111/nph.16468 32017125

[nph17325-bib-0036] Liao C , Zheng Y , Guo Y . 2017. MYB30 transcription factor regulates oxidative and heat stress responses through ANNEXIN‐mediated cytosolic calcium signaling in *Arabidopsis* . New Phytologist 216: 163–177. 10.1111/nph.14679 28726305

[nph17325-bib-0037] Lim A , Zhang L . 1999. WebPHYLIP: a web interface to PHYLIP. Bioinformatics 15: 1068–1069. 10746002 10.1093/bioinformatics/15.12.1068

[nph17325-bib-0038] Liu H , Luo X , Niu L , Xiao Y , Chen L , Liu J , Wang X , Jin M , Li W , Zhang Q *et al*. 2017. Distant eQTLs and non‐coding sequences play critical roles in regulating gene expression and quantitative trait variation in maize. Molecular Plant 10: 414–426. 27381443 10.1016/j.molp.2016.06.016

[nph17325-bib-0039] Liu S , Li C , Wang H , Wang S , Yang S , Liu X , Yan J , Li B , Beatty M , Zastrow‐Hayes G *et al*. 2020. Mapping regulatory variants controlling gene expression in drought response and tolerance in maize. Genome Biology 21: 163. 32631406 10.1186/s13059-020-02069-1 PMC7336464

[nph17325-bib-0040] Lobell DB , Roberts MJ , Schlenker W , Braun N , Little BB , Rejesus RM , Hammer GL . 2014. Greater sensitivity to drought accompanies maize yield increase in the U.S. Midwest. Science 344: 516–519. 24786079 10.1126/science.1251423

[nph17325-bib-0041] Ma H . 2005. Molecular genetic analyses of microsporogenesis and microgametogenesis in flowering plants. Annual Review of Plant Biology 56: 393–434. 10.1146/annurev.arplant.55.031903.141717 15862102

[nph17325-bib-0042] Ma Y , Min L , Wang M , Wang C , Zhao Y , Li Y , Fang Q , Wu Y , Xie S , Ding Y *et al*. 2018. Disrupted genome methylation in response to high temperature has distinct affects on microspore abortion and anther indehiscence. Plant Cell 30: 1387–1403. 29866646 10.1105/tpc.18.00074 PMC6096589

[nph17325-bib-0043] Ma Z , He S , Wang X , Sun J , Zhang Y , Zhang G , Wu L , Li Z , Liu Z , Sun G *et al*. 2018. Resequencing a core collection of upland cotton identifies genomic variation and loci influencing fiber quality and yield. Nature Genetics 50: 803–813. 29736016 10.1038/s41588-018-0119-7

[nph17325-bib-0044] Mao H , Wang H , Liu S , Li Z , Yang X , Yan J , Li J , Tran LS , Qin F . 2015. A transposable element in a *NAC* gene is associated with drought tolerance in maize seedlings. Nature Communications 6: 8326. 10.1038/ncomms9326 PMC4595727 26387805

[nph17325-bib-0045] Min L , Li Y , Hu Q , Zhu L , Gao W , Wu Y , Ding Y , Liu S , Yang X , Zhang X . 2014. Sugar and auxin signaling pathways respond to high‐temperature stress during anther development as revealed by transcript profiling analysis in cotton. Plant Physiology 164: 1293–1308. 24481135 10.1104/pp.113.232314 PMC3938621

[nph17325-bib-0046] Min L , Zhu L , Tu L , Deng F , Yuan D , Zhang X . 2013. Cotton *GhCKI* disrupts normal male reproduction by delaying tapetum programmed cell death via inactivating starch synthase. The Plant Journal 75: 823–835. 23662698 10.1111/tpj.12245

[nph17325-bib-0047] Pettigrew WT . 2008. The effect of higher temperatures on cotton lint yield production and fiber quality. Crop Science 48: 278–285.

[nph17325-bib-0048] Poland JA , Bradbury PJ , Buckler ES , Nelson RJ . 2011. Genome‐wide nested association mapping of quantitative resistance to northern leaf blight in maize. Proceedings of the National Academy of Sciences, USA 108: 6893–6898. 10.1073/pnas.1010894108 PMC3084105 21482771

[nph17325-bib-0049] Purcell S , Neale B , Todd‐Brown K , Thomas L , Ferreira MAR , Bender D , Maller J , Sklar P , de Bakker PIW , Daly MJ *et al*. 2007. PLINK: a tool set for whole‐genome association and population‐based linkage analyses. American Journal of Human Genetics 81: 559–575. 17701901 10.1086/519795 PMC1950838

[nph17325-bib-0050] Ranjan A , Budke JM , Rowland SD , Chitwood DH , Kumar R , Carriedo L , Ichihashi Y , Zumstein K , Maloof JN , Sinha NR . 2016. eQTL regulating transcript levels associated with diverse biological processes in tomato. Plant Physiology 172: 328–340. 27418589 10.1104/pp.16.00289 PMC5074602

[nph17325-bib-0051] Sakata T , Oshino T , Miura S , Tomabechi M , Tsunaga Y , Higashitani N , Miyazawa Y , Takahashi H , Watanabe M , Higashitani A . 2010. Auxins reverse plant male sterility caused by high temperatures. Proceedings of the National Academy of Sciences, USA 107: 8569–8574. 10.1073/pnas.1000869107 PMC2889339 20421476

[nph17325-bib-0052] Schauberger B , Archontoulis S , Arneth A , Balkovic J , Ciais P , Deryng D , Elliott J , Folberth C , Khabarov N , Muller C *et al*. 2017. Consistent negative response of US crops to high temperatures in observations and crop models. Nature Communications 8: 13931. 10.1038/ncomms13931 PMC5253679 28102202

[nph17325-bib-0053] Seneviratne SI , Rogelj J , Séférian R , Wartenburger R , Allen MR , Cain M , Millar RJ , Ebi KL , Ellis N , Hoegh‐Guldberg O *et al*. 2018. The many possible climates from the Paris Agreement’s aim of 1.5 °C warming. Nature 558: 41–49. 29875489 10.1038/s41586-018-0181-4

[nph17325-bib-0054] Shabalin AA . 2012. Matrix eQTL: ultra fast eQTL analysis via large matrix operations. Bioinformatics 28: 1353–1358. 22492648 10.1093/bioinformatics/bts163 PMC3348564

[nph17325-bib-0081] Shen C , Wang N , Huang C , Wang WJ , Zhang XL . 2019. Population genomics reveals a fine‐scale recombination landscape for genetic improvement of cotton. The Plant Journal 99: 494–505. 31002209 10.1111/tpj.14339

[nph17325-bib-0055] Shen H , Zhong X , Zhao F , Wang Y , Yan B , Li Q , Chen G , Mao B , Wang J , Li Y *et al*. 2015. Overexpression of receptor‐like kinase *ERECTA* improves thermotolerance in rice and tomato. Nature Biotechnology 33: 996–1003. 10.1038/nbt.3321 26280413

[nph17325-bib-0056] Shen S , Park JW , Lu Z‐x , Lin L , Henry MD , Wu YN , Zhou Q , Xing Y . 2014. rMATS: Robust and flexible detection of differential alternative splicing from replicate RNA‐Seq data. Proceedings of the National Academy of Sciences, USA 111: E5593–E5601. 10.1073/pnas.1419161111 PMC4280593 25480548

[nph17325-bib-0057] Si L , Chen J , Huang X , Gong H , Luo J , Hou Q , Zhou T , Lu T , Zhu J , Shangguan Y *et al*. 2016. *OsSPL13* controls grain size in cultivated rice. Nature Genetics 48: 447–456. 26950093 10.1038/ng.3518

[nph17325-bib-0058] Silva IT , Rosales RA , Holanda AJ , Nussenzweig MC , Jankovic M . 2014. Identification of chromosomal translocation hotspots via scan statistics. Bioinformatics 30: 2551–2558. 24860160 10.1093/bioinformatics/btu351 PMC4155254

[nph17325-bib-0059] Stromme CB , Julkunen‐Tiitto R , Krishna U , Lavola A , Olsen JE , Nybakken L . 2015. UV‐B and temperature enhancement affect spring and autumn phenology in *Populus tremula* . Plant, Cell & Environment 38: 867–877. 10.1111/pce.12338 24689776

[nph17325-bib-0060] Tang S , Zhao H , Lu S , Yu L , Zhang G , Zhang Y , Yang QY , Zhou Y , Wang X , Ma W *et al*. 2020. Genome‐ and transcriptome‐wide association studies provide insights into the genetic basis of natural variation of seed oil content in *Brassica napus* . Molecular Plant 14: 470–487. 33309900 10.1016/j.molp.2020.12.003

[nph17325-bib-0061] Teng C , Zhang H , Hammond R , Huang K , Meyers BC , Walbot V . 2020. *Dicer‐like 5* deficiency confers temperature‐sensitive male sterility in maize. Nature Communications 11: 2912. 10.1038/s41467-020-16634-6 PMC7283321 32518237

[nph17325-bib-0062] Wainberg M , Sinnott‐Armstrong N , Mancuso N , Barbeira AN , Knowles DA , Golan D , Ermel R , Ruusalepp A , Quertermous T , Hao K *et al*. 2019. Opportunities and challenges for transcriptome‐wide association studies. Nature Genetics 51: 592–599. 30926968 10.1038/s41588-019-0385-z PMC6777347

[nph17325-bib-0063] Wang J , Yu H , Xie W , Xing Y , Yu S , Xu C , Li X , Xiao J , Zhang Q . 2010. A global analysis of QTLs for expression variations in rice shoots at the early seedling stage. The Plant Journal 63: 1063–1074. 20626655 10.1111/j.1365-313X.2010.04303.x

[nph17325-bib-0064] Wang M , Tu L , Lin M , Lin Z , Wang P , Yang Q , Ye Z , Shen C , Li J , Zhang L *et al*. 2017. Asymmetric subgenome selection and *cis*‐regulatory divergence during cotton domestication. Nature Genetics 49: 579–587. 28263319 10.1038/ng.3807

[nph17325-bib-0065] Wang M , Tu L , Yuan D , Zhu D , Shen C , Li J , Liu F , Pei L , Wang P , Zhao G *et al*. 2019. Reference genome sequences of two cultivated allotetraploid cottons, *Gossypium hirsutum* and *Gossypium barbadense* . Nature Genetics 51: 224–229. 30510239 10.1038/s41588-018-0282-x

[nph17325-bib-0066] Wang M , Yuan D , Tu L , Gao W , He Y , Hu H , Wang P , Liu N , Lindsey K , Zhang X . 2015. Long noncoding RNAs and their proposed functions in fibre development of cotton (*Gossypium* spp.). New Phytologist 207: 1181–1197. 10.1111/nph.13429 25919642

[nph17325-bib-0067] Wang X , Chen Q , Wu Y , Lemmon ZH , Xu G , Huang C , Liang Y , Xu D , Li D , Doebley JF *et al*. 2018. Genome‐wide analysis of transcriptional variability in a large maize‐teosinte population. Molecular Plant 11: 443–459. 29275164 10.1016/j.molp.2017.12.011

[nph17325-bib-0068] Wu D , Liang Z , Yan T , Xu Y , Xuan L , Tang J , Zhou G , Lohwasser U , Hua S , Wang H *et al*. 2019. Whole‐genome resequencing of a worldwide collection of rapeseed accessions reveals the genetic basis of ecotype divergence. Molecular Plant 12: 30–43. 30472326 10.1016/j.molp.2018.11.007

[nph17325-bib-0069] Wu L , Shi W , Long J , Guo X , Michailidou K , Beesley J , Bolla MK , Shu XO , Lu Y , Cai Q *et al*. 2018. A transcriptome‐wide association study of 229,000 women identifies new candidate susceptibility genes for breast cancer. Nature Genetics 50: 968–978. 29915430 10.1038/s41588-018-0132-x PMC6314198

[nph17325-bib-0070] Wu Y , Min L , Wu Z , Yang L , Zhu L , Yang X , Yuan D , Guo X , Zhang X . 2015. Defective pollen wall contributes to male sterility in the male sterile line 1355A of cotton. Scientific Reports 5: 9608. 26043720 10.1038/srep09608 PMC4456728

[nph17325-bib-0071] Wunderlich M , Gross‐Hardt R , Schoffl F . 2014. Heat shock factor HSFB2a involved in gametophyte development of *Arabidopsis thaliana* and its expression is controlled by a heat‐inducible long non‐coding antisense RNA. Plant Molecular Biology 85: 541–550. 24874772 10.1007/s11103-014-0202-0 PMC4099531

[nph17325-bib-0072] Xu Y , Zhang L , Ou S , Wang R , Wang Y , Chu C , Yao S . 2020. Natural variations of *SLG1* confer high‐temperature tolerance in indica rice. Nature Communications 11: 5441. 10.1038/s41467-020-19320-9 PMC7595236 33116138

[nph17325-bib-0073] Zahid KR , Ali F , Shah F , Younas M , Shah T , Shahwar D , Hassan W , Ahmad Z , Qi C , Lu Y *et al*. 2016. Response and tolerance mechanism of cotton *Gossypium hirsutum* L. to elevated temperature stress: a review. Frontiers in Plant Science 7: 937. 27446165 10.3389/fpls.2016.00937 PMC4927942

[nph17325-bib-0074] Zhang L , Su W , Tao R , Zhang W , Chen J , Wu P , Yan C , Jia Y , Larkin RM , Lavelle D *et al*. 2017. RNA sequencing provides insights into the evolution of lettuce and the regulation of flavonoid biosynthesis. Nature Communications 8: 2264. 10.1038/s41467-017-02445-9 PMC5741661 29273740

[nph17325-bib-0075] Zhang L , Wang M , Li N , Wang H , Qiu P , Pei L , Xu Z , Wang T , Gao E , Liu J *et al*. 2018. Long noncoding RNAs involve in resistance to *Verticillium dahliae*, a fungal disease in cotton. Plant Biotechnology Journal 16: 1172–1185. 29149461 10.1111/pbi.12861 PMC5978870

[nph17325-bib-0076] Zhao B , Shi H , Wang W , Liu X , Gao H , Wang X , Zhang Y , Yang M , Li R , Guo Y . 2016. Secretory COPII protein SEC31B is required for pollen wall development. Plant Physiology 172: 1625–1642. 27634427 10.1104/pp.16.00967 PMC5100771

[nph17325-bib-0077] Zhao C , Liu B , Piao S , Wang X , Lobell DB , Huang Y , Huang M , Yao Y , Bassu S , Ciais P *et al*. 2017. Temperature increase reduces global yields of major crops in four independent estimates. Proceedings of the National Academy of Sciences, USA 114: 9326–9331. 10.1073/pnas.1701762114 PMC5584412 28811375

[nph17325-bib-0078] Zhao T , Tao X , Feng S , Wang L , Hong H , Ma W , Shang G , Guo S , He Y , Zhou B *et al*. 2018. LncRNAs in polyploid cotton interspecific hybrids are derived from transposon neofunctionalization. Genome Biology 19: 195. 30419941 10.1186/s13059-018-1574-2 PMC6233382

[nph17325-bib-0079] Zhou Z , Jiang Y , Wang Z , Gou Z , Lyu J , Li W , Yu Y , Shu L , Zhao Y , Ma Y *et al*. 2015. Resequencing 302 wild and cultivated accessions identifies genes related to domestication and improvement in soybean. Nature Biotechnology 33: 408–414. 10.1038/nbt.3096 25643055

[nph17325-bib-0080] Zhu J , Lou Y , Shi QS , Zhang S , Zhou WT , Yang J , Zhang C , Yao XZ , Xu T , Liu JL *et al*. 2020. Slowing development restores the fertility of thermo‐sensitive male‐sterile plant lines. Nature Plants 6: 360–367. 32231254 10.1038/s41477-020-0622-6

