# Supplementary material for: A combination of genome‐wide and transcriptome‐wide association studies reveals genetic elements leading to male sterility during high temperature stress in cotton
Source: New Phytol. 2021 May 2;231(1):165–81. doi: 10.1111/nph.17325 (PMC8252431; doi:10.1111/nph.17325)

New Phytologist Supporting Information:

Combined transcriptome GWAS and TWAS reveal genetic elements leading to male sterility during high temperature stress in cotton

Yizan Ma, Ling Min, Junduo Wang, Yaoyao Li, Yuanlong Wu, Qin Hu, Yuanhao Ding, Maojun Wang, Yajun Liang, Zhaolong Gong, Sai Xie, Xiaojun Su, Chaozhi Wang, Yunlong Zhao, Qidi Fang, Yanlong Li, Huabin Chi, Miao Chen, Aamir Hamid Khan, Keith Lindsey, Longfu Zhu, Xueyuan Li, Xianlong Zhang
The article acceptance date: 23 February 2021

Supplemental Figures

Fig. S1 Images of field in Alear **(a)**, Wuhan **(b)**, Turpan **(c)** and greenhouse in Wuhan **(d)**.
Fig. S2 Analysis of positions and potential functions of SNPs.
Fig. S3 Analysis of Evanno's △*K* from STRUCTURE results.
Fig. S4 Detailed information of PCA of three subpopulations.
Fig. S5 Linkage disequilibrium decay rate in At and Dt subgenomes.
Fig. S6 Hierarchical clustering of all accessions.
Fig. S7 Proportion of different kinds of transposable elements (TEs) in the genome.
Fig. S8 Analysis of network topology using different soft-thresholding powers.
Fig. S9 Module classification and correlation analysis of each module.
Fig. S10 Heatmap of expression profile of 15 modules.
Fig. S11 Gene Ontology analysis of genes in 'black' module.
Fig. S12 Expression levels of putative associated genes in different genotype accessions.
Fig. S13 Transcriptome-wide association study based on expression imputation with *cis*-SNPs.
Fig. S14 Association analysis in significant intervals in D01 **(a)** and D05 **(b)** chromosomes.
Fig. S15 Protein domain analysis of *Ghir_A01G006180, At4g27290* and *GH_A01G0682*.
Fig. S16 The coverage of sequencing reads for *Ghir_A01G006180* in eight accessions with different phenotypes.
Fig. S17 Nucleic acid sequence alignment of *Ghir_A01G006180, GH_A01G0682* and *GH_A01G0683*.
Fig. S18 Functional annotation of 13 significantly associated SNPs in *GhHRK1*.
Fig. S19 Differentially expressed MYB transcription factors in accessions with distinct phenotype.
Fig. S20 Pollen viability images of accessions selected to perform in situ hybridization.
Fig. S21 In situ hybridization of *GhHRK1* at tapetum degradation stage for the same accessions as in Fig. 5.
Fig. S22 Phylogenetic analysis of *GhHRK1* and transcripts of G-type lectin protein kinase in *Arabidopsis*.
Fig. S23 An overview image of vegetative development of wild type (WT) and two *hrk1* mutant lines during seedling period.
Fig. S24 Pollen viability of WT and two *hrk1* mutants under NT control and HT stress.
Fig. S25 Images of siliques and inflorescence of WT, *hrk1-1* and *hrk1-2* after HT treatment.
Fig. S26 Transgenic complement assay of *GhHRK1* in *Arabidopsis*.
Fig. S27 Phenotypic analysis of *GhHRK1* transgenic individuals under NT **(a)** and HT. **(b)**.

Fig. S1 Images of field in Alear (**a**), Wuhan (**b**), Turpan (**c**) and greenhouse in Wuhan (**d**).

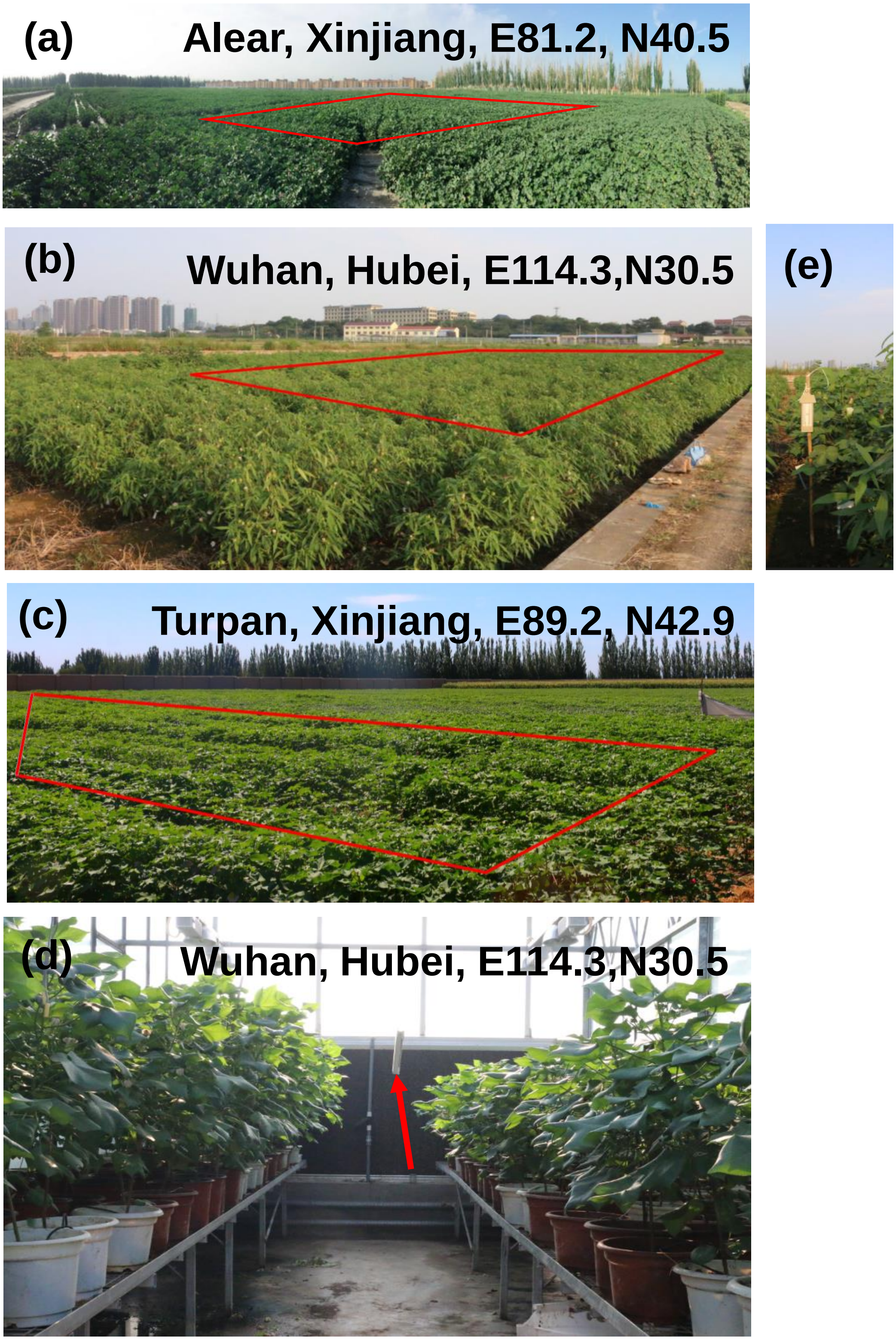

The thermometers were set in the field (**e**) and greenhouse (red arrow in **d**) to monitor the highest temperature. The experimental blocks were indicated by red squares in the figures.

Fig. S2 Analysis of positions and potential functions of SNPs.

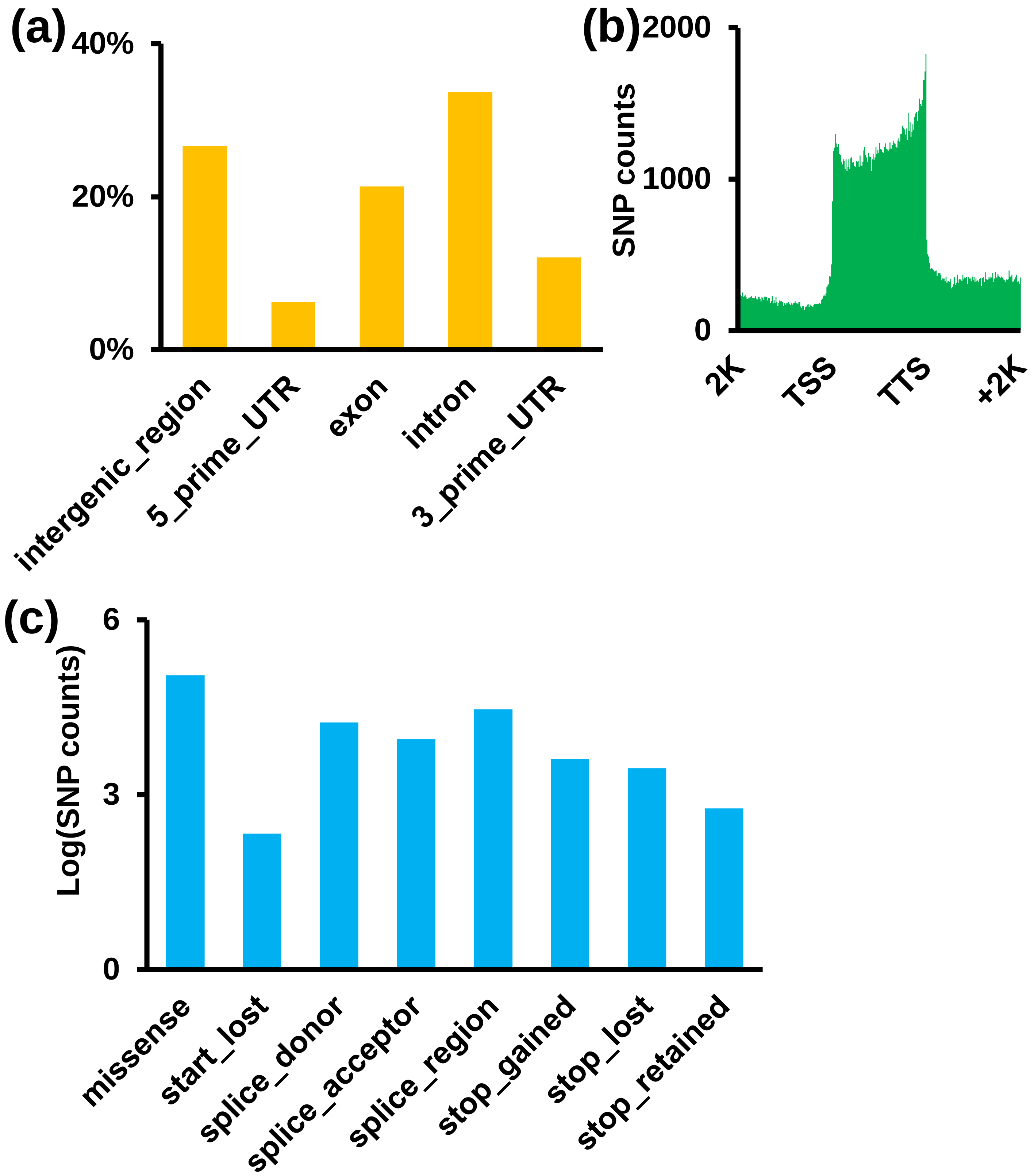

(a) SNPs were relatively enriched in the intergenic and intron regions.
(b) SNPs located in the genic regions are abundant in the gene body. TSS, transcription start site; TTS, transcription termination site.
(c) Number of functional SNPs in gene regions.

Fig. S3 Analysis of Evanno's $\triangle K$ from STRUCTURE results.

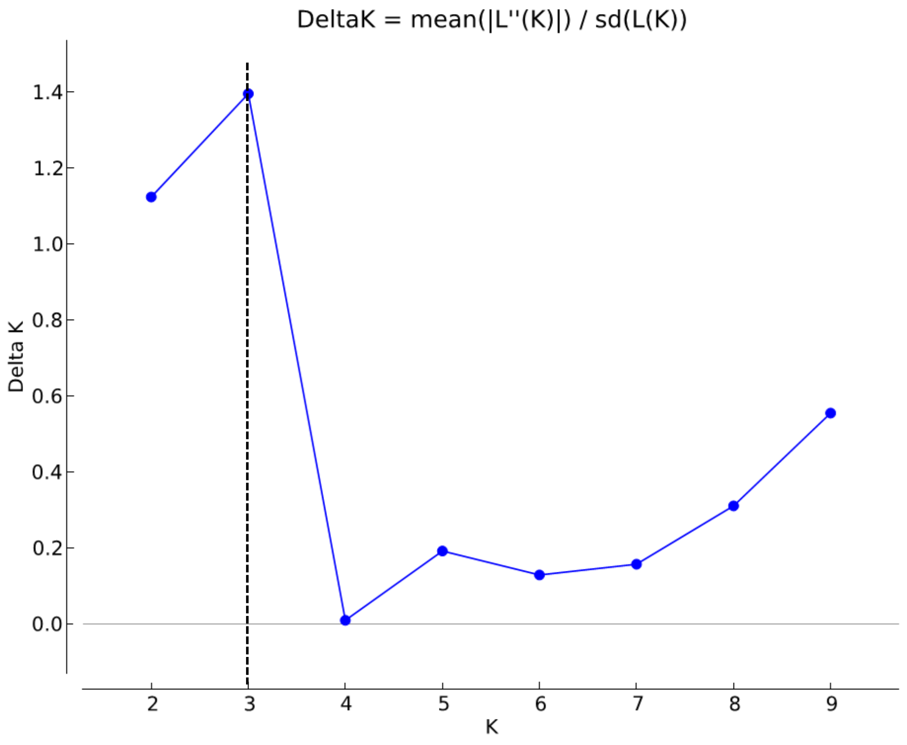

The value of Evanno's $\triangle K$ showed a clear spike at K = 3. The vertical dashed line suggested the population can be divided into 3 subpopulations.

Fig. S4 Detailed information of PCA of three subpopulations.

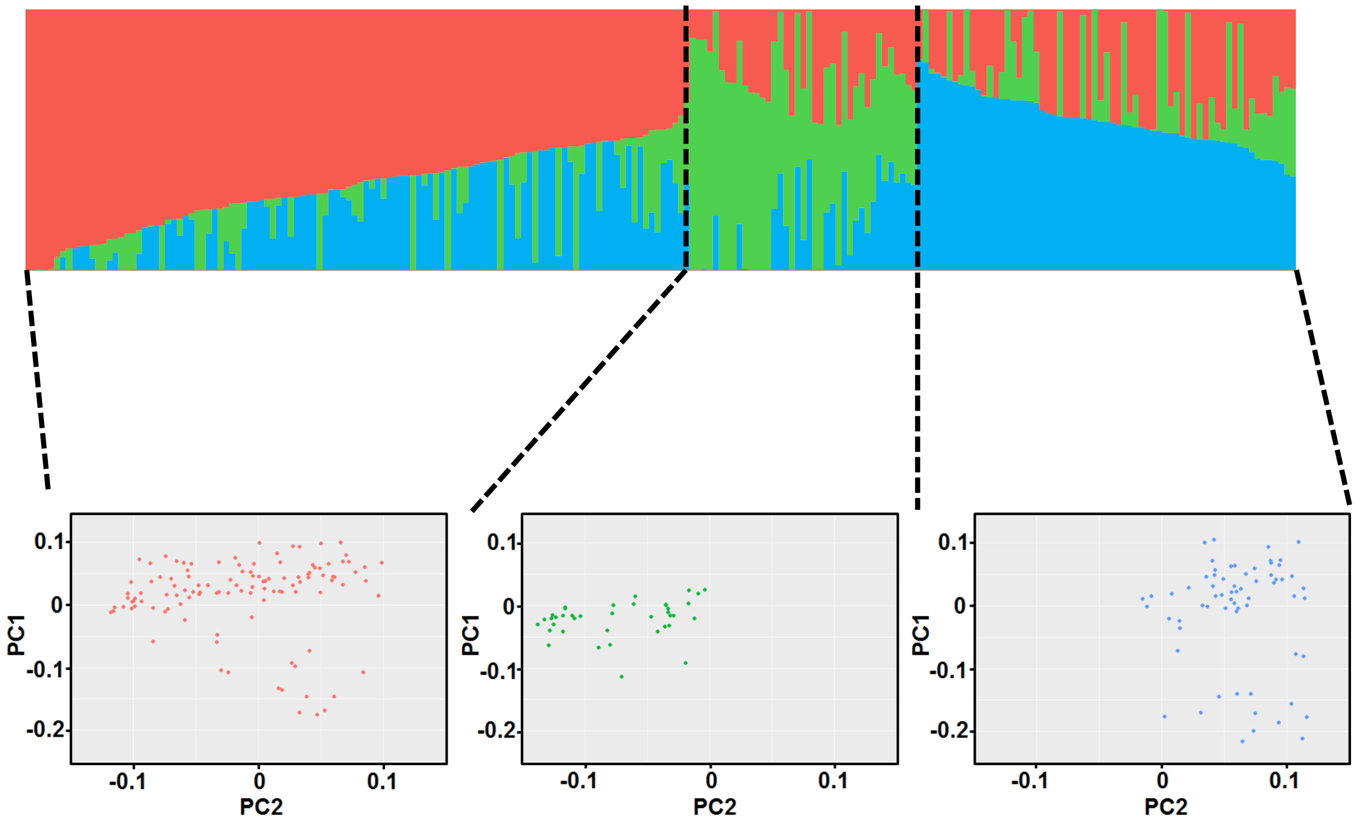

Accessions classified into green and blue subpopulations can be divided. The accessions in the red group covered the accessions in green and blue subpopulations

Fig. S5 Linkage disequilibrium decay rate in At and Dt subgenomes.

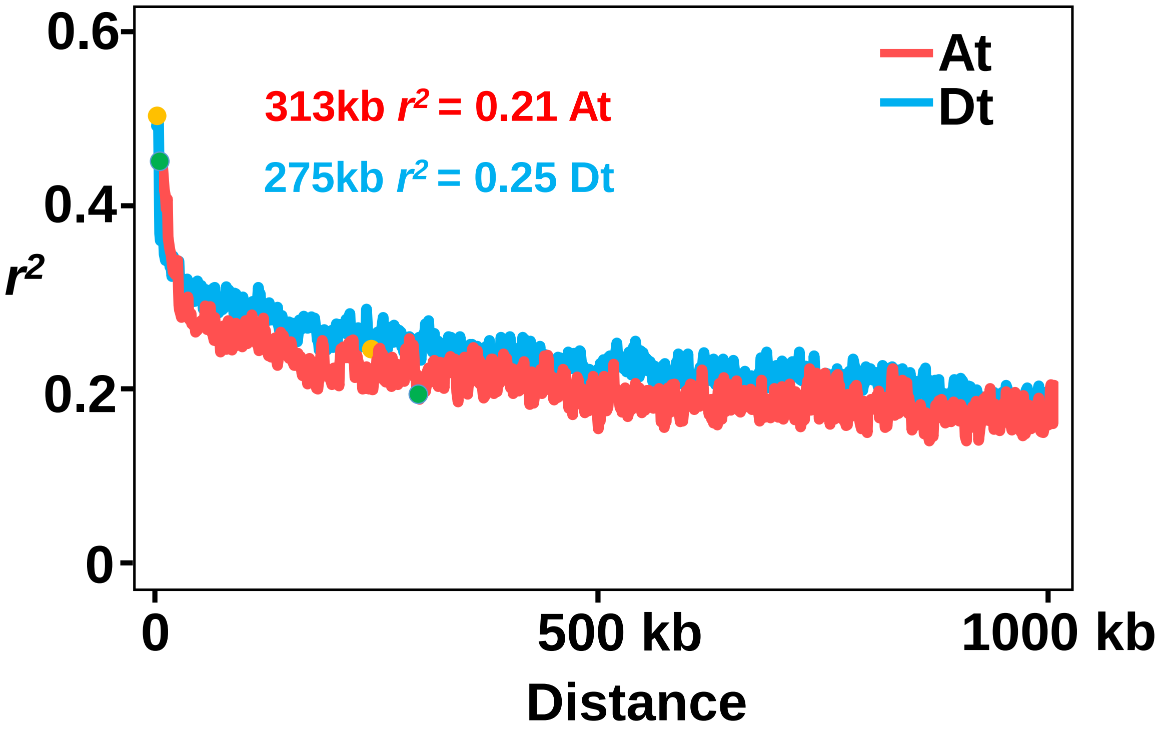

The distance was estimated to be 313 kb (r^2 = 0.21) and 275 kb (r^2 = 0.25) in At and Dt subgenomes respectively.

Fig. S6 Hierarchical clustering of all accessions.

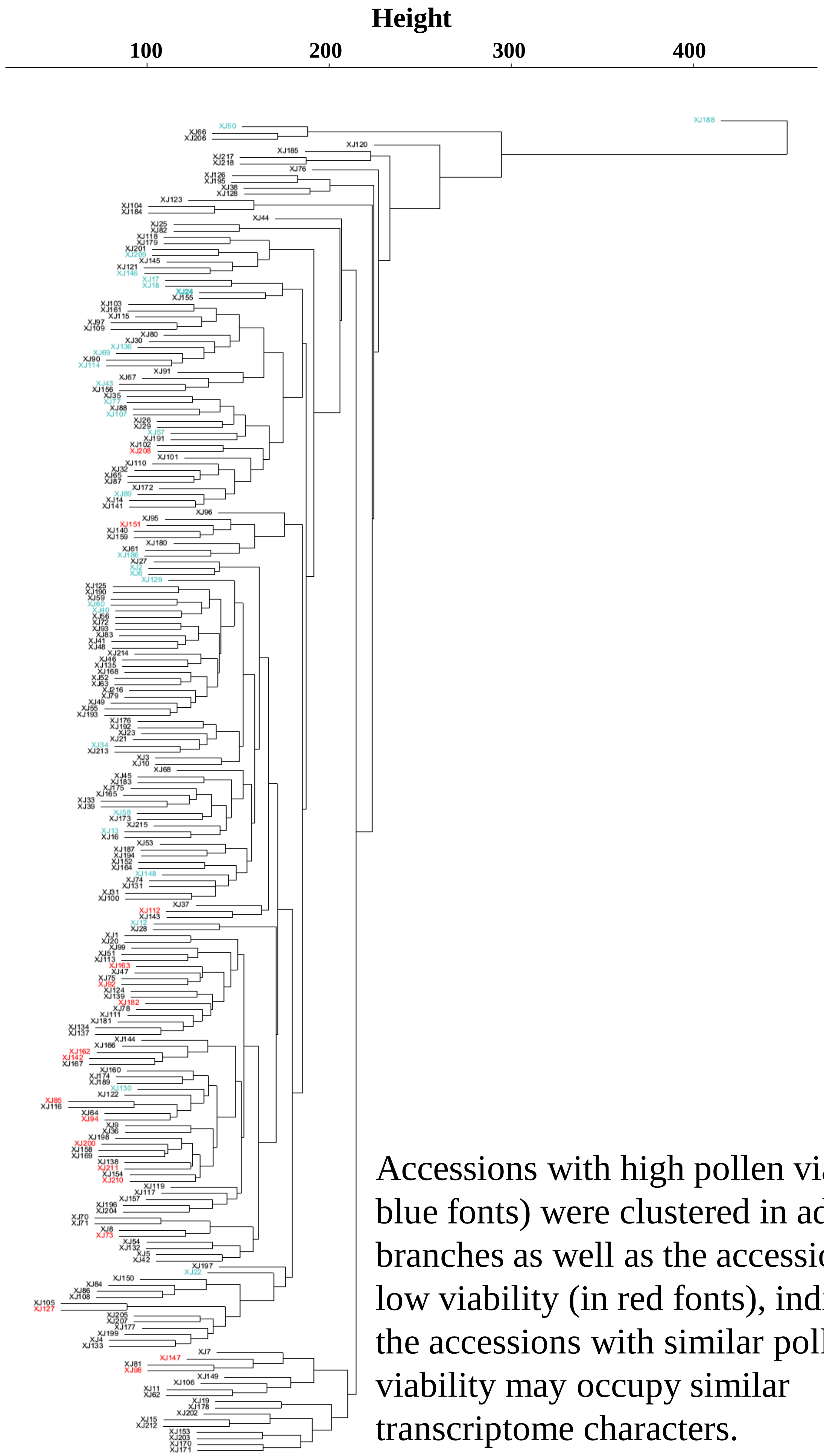

Accessions with high pollen viability (in blue fonts) were clustered in adjacent branches as well as the accessions with low viability (in red fonts), indicating the accessions with similar pollen viability may occupy similar transcriptome characters.

Fig. S7 Proportions of different kinds of transposable elements (TEs) in the genome.

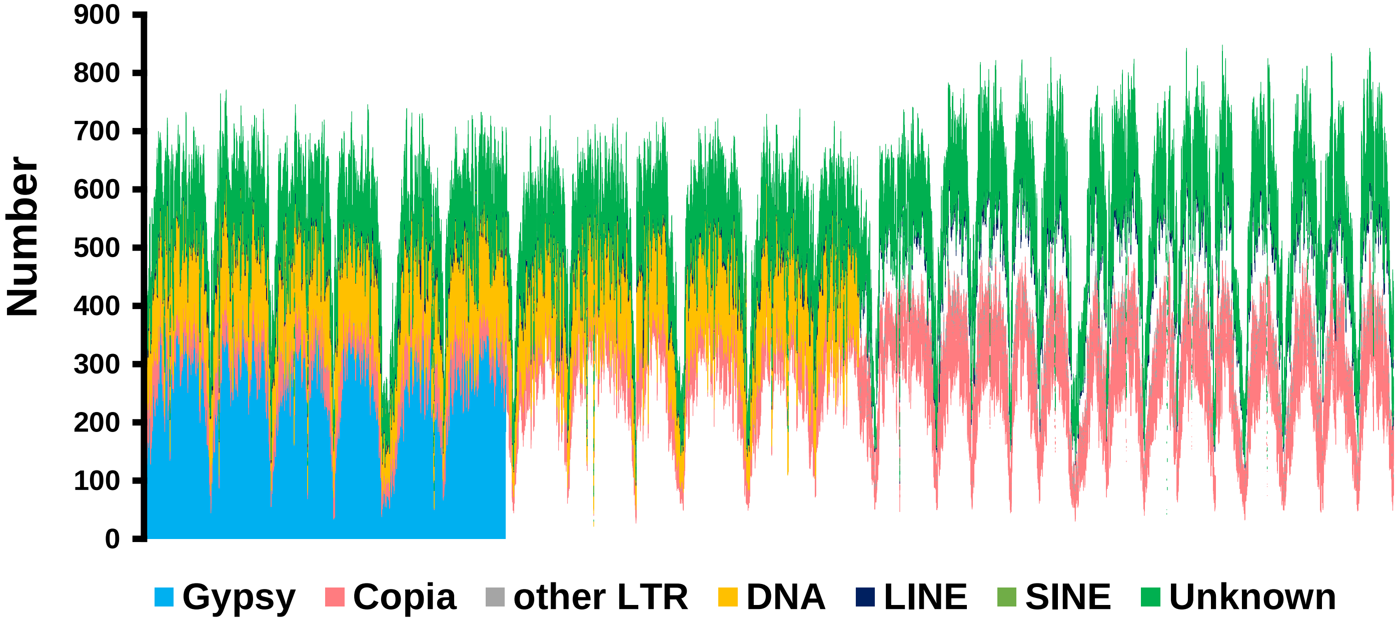

Long terminal repeats (LTR) including Gypsy and Copia represented a large proportion of TEs (more than 50%) in the genome. The *y*-axis denoted the number of TEs in the separated genomic regions.

Fig. S8 Analysis of network topology using different soft-thresholding powers.

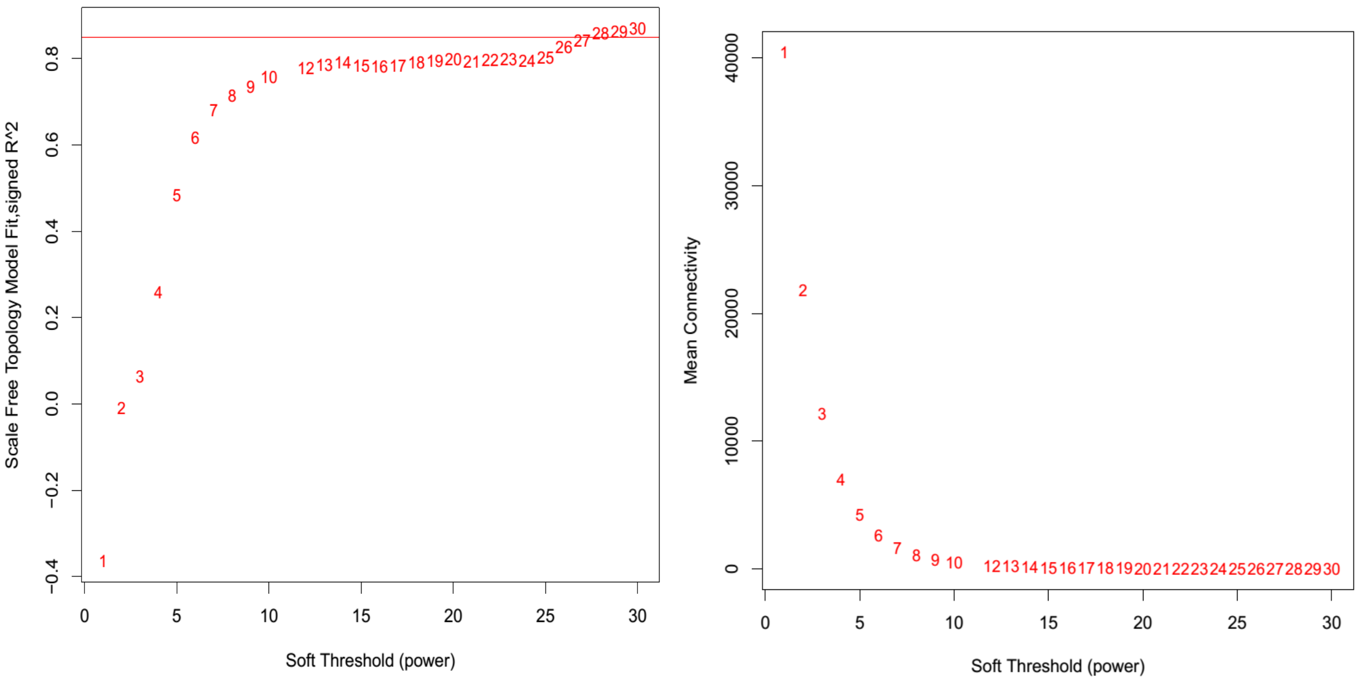

The left panel showed the scale-free fit index (*y*-axis) as a function of the soft-thresholding power (*x*-axis). The red line indicated 0.85 cutoff in the plot. The right panel showed the mean connectivity (*y*-axis) as a function of the soft-thresholding power (*x*-axis). Soft-thresholding power 28 was chosen for network construction.

Fig. S9 Module classification and correlation analysis of each module.

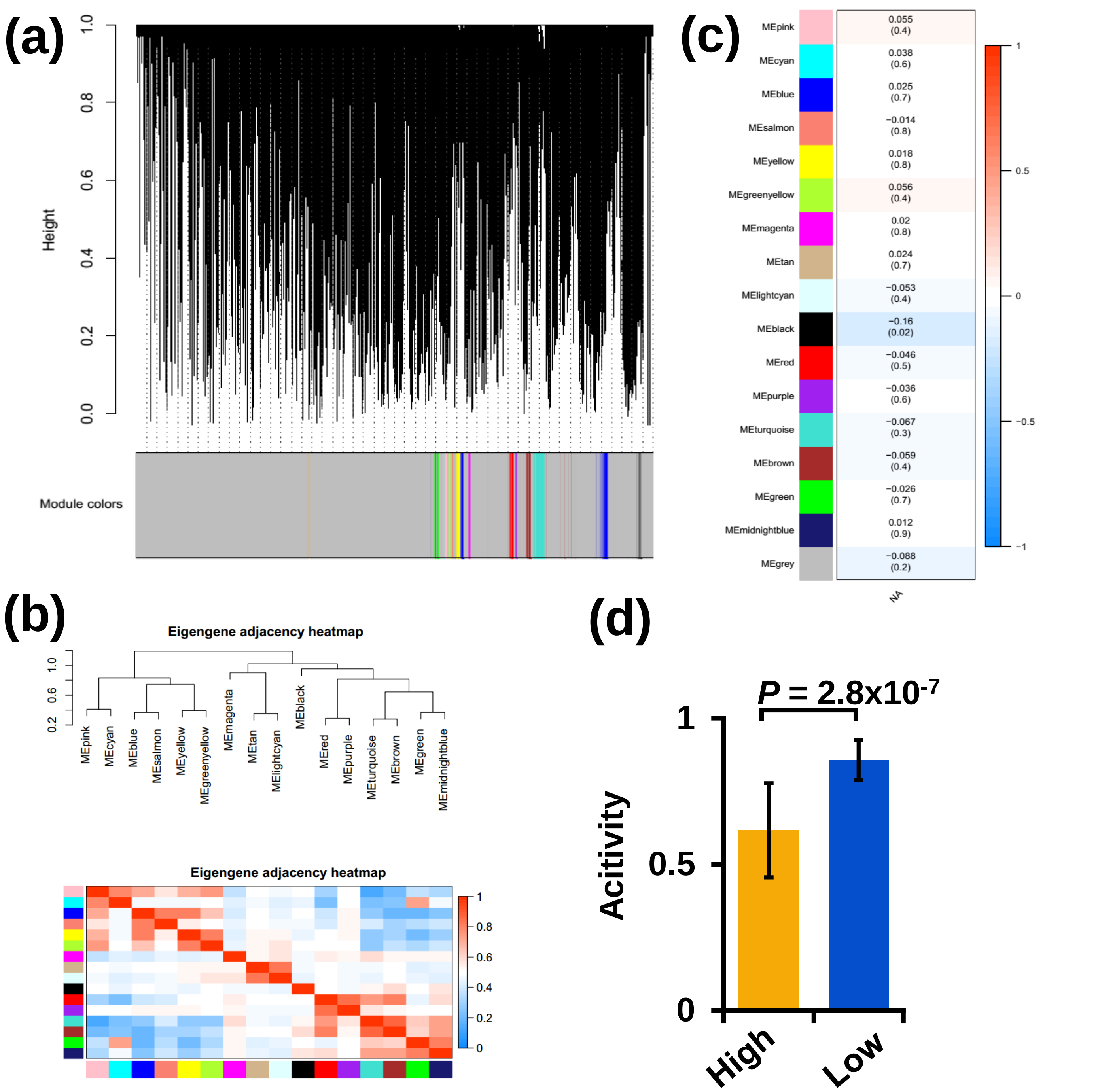

(a) Network containing 16 modules, with different colors representing different modules. Genes which classified in grey scales were not presented in any other modules.
(b) Adjacency analysis between different modules. The heatmap corresponded to the adjacency tree. The modules in adjacent tree branches showed higher adjacencies which were displayed in the heatmap.
(c) Pearson correlation analysis between pollen viability and each module. The correlation coefficient (*r^2* upon bracket) and significant check (*P*-value in bracket) were shown in the plot. Genes in the 'black' module showed significant correlation with pollen viability.
(d) Accessions with increased expression trends of genes in the 'black' module displayed lower pollen viability (shown in yellow column). The right blue column showed 110 accessions with low expressions of genes in the 'black' module. Significant difference was found between the two groups (two-tailed Student's *t*-test). The values were mean ± SD

Fig. S10 Heatmap of expression profile of 15 modules. Genes different modules showed totally different expression pattern. (contained 4 pages)

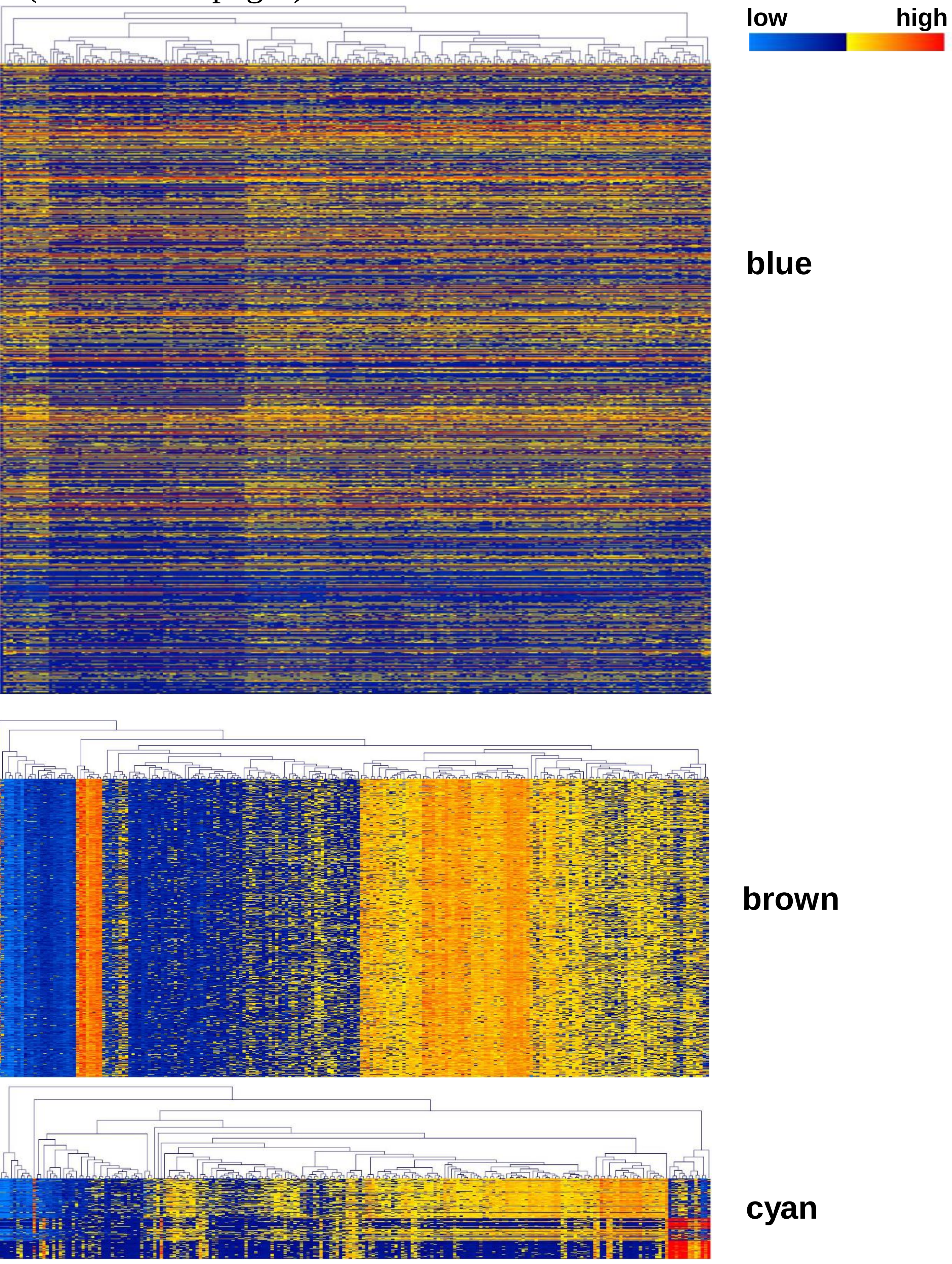

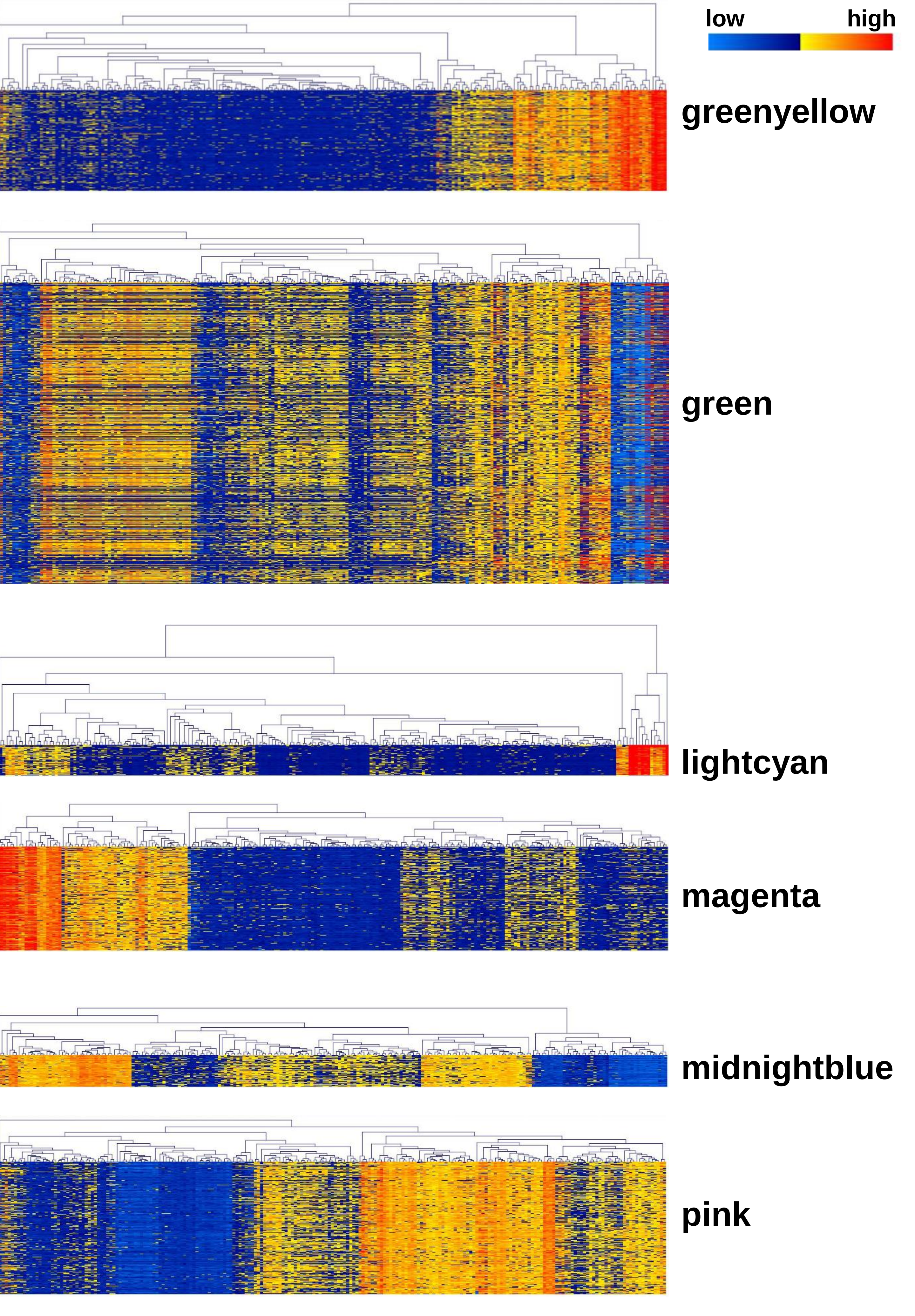
low
high
greenyellow
green
lightcyan
magenta
midnightblue
pink

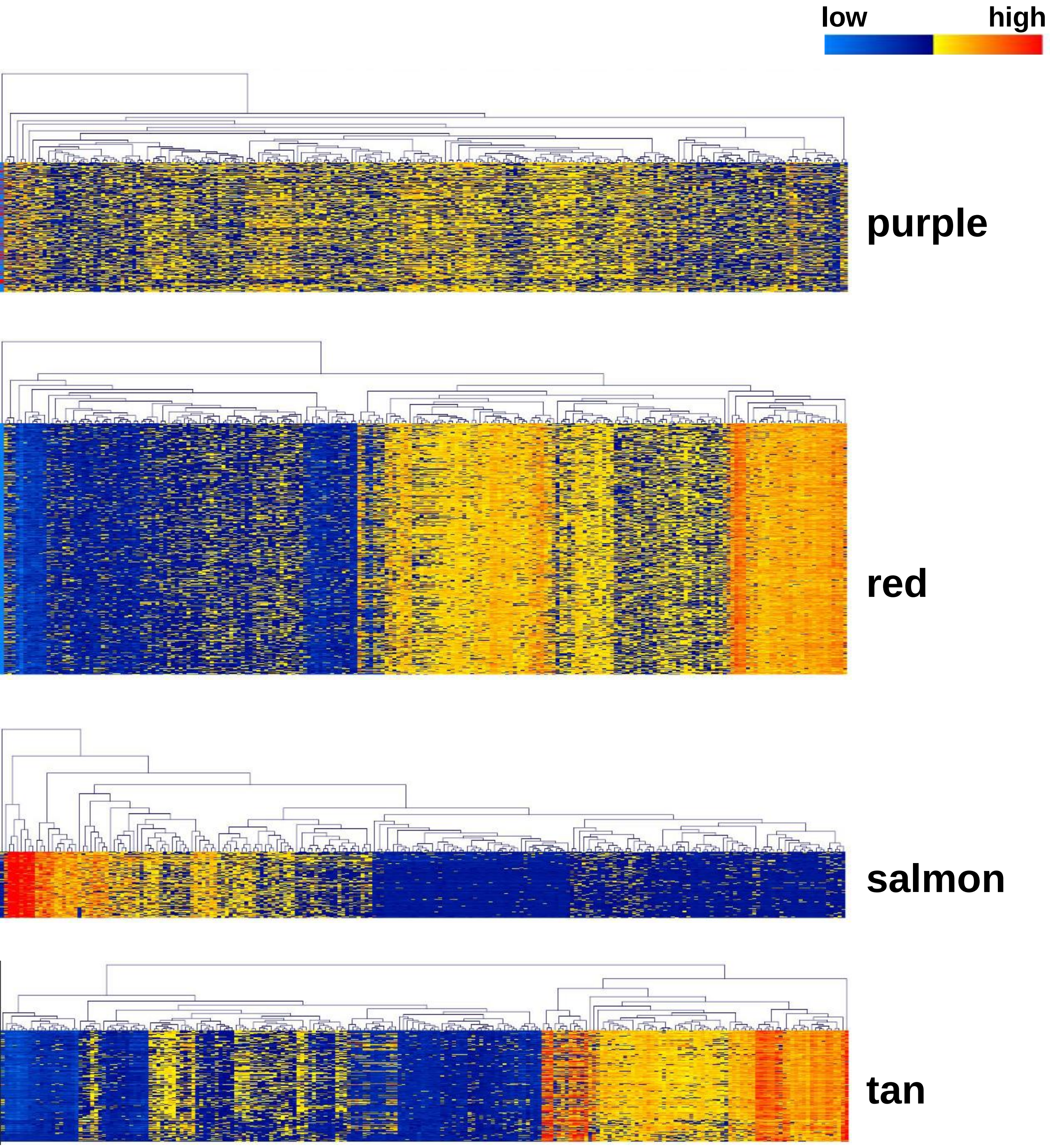
low
high
purple
red
salmon
tan

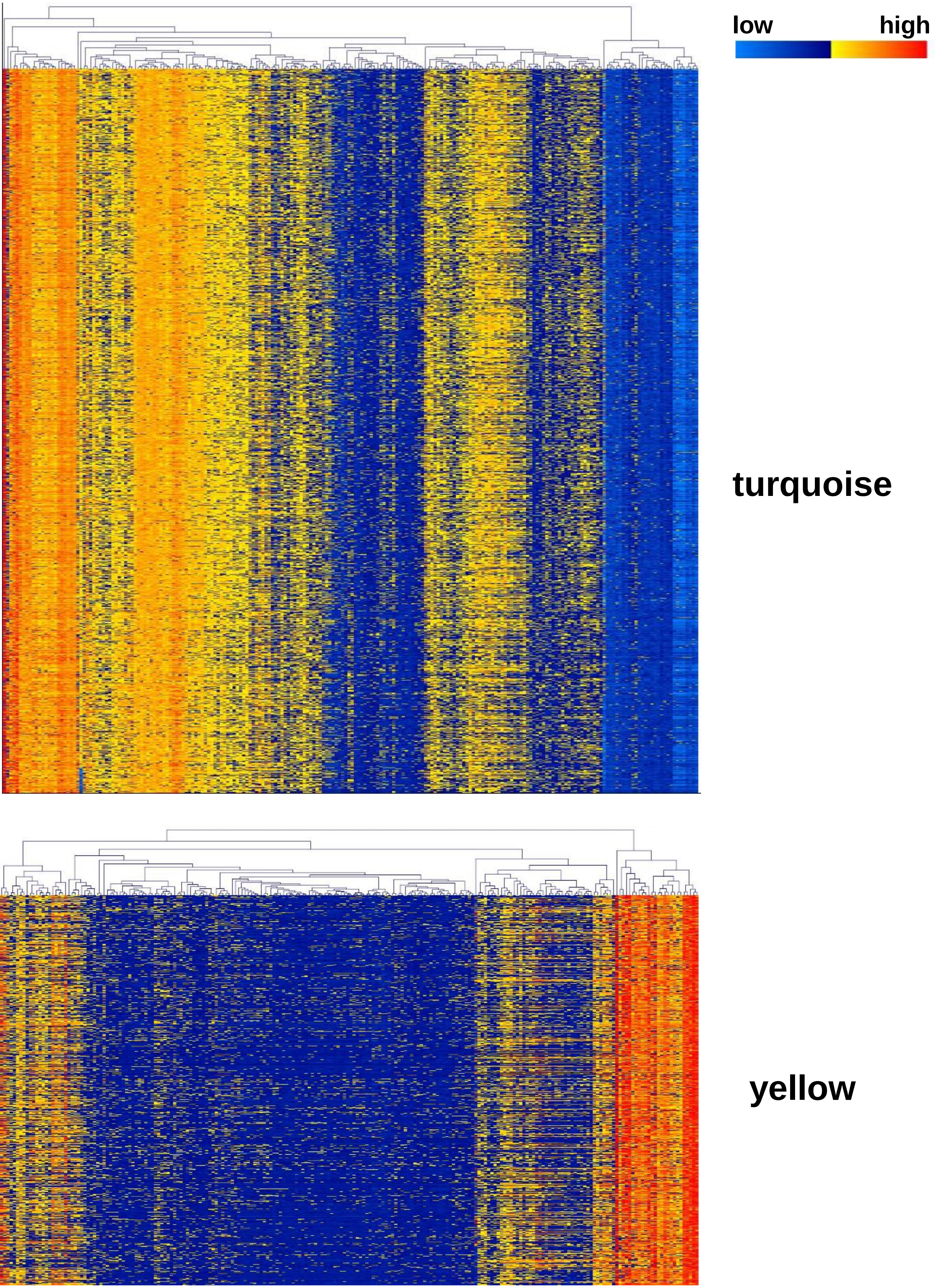
low
high
turquoise
yellow

Fig. S11 Gene Ontology analysis of genes in black module.

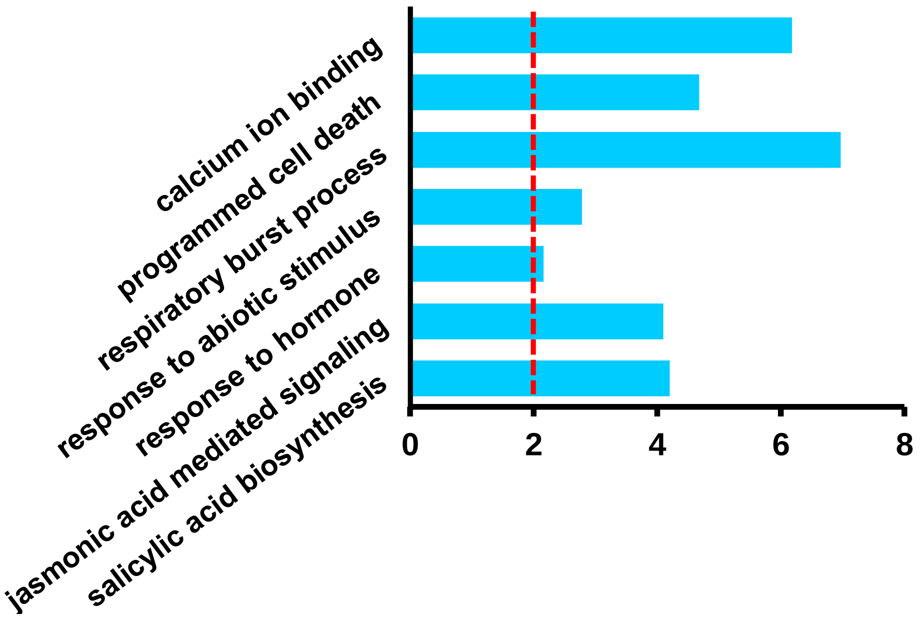

The *P*-value 0.01 was set as significant cutoff. Genes participating in pivotal cell process were enriched in the 'black' module.

Fig. S12 Expression levels of putative associated genes in different genotype accessions. The comparison was conducted using unpaired *t*-test. The difference of expression levels were indicated by *P*-value among different genotypes. The *y*-axis indicated the FPKM values of corresponding genes. The outliers were shown as black dots.

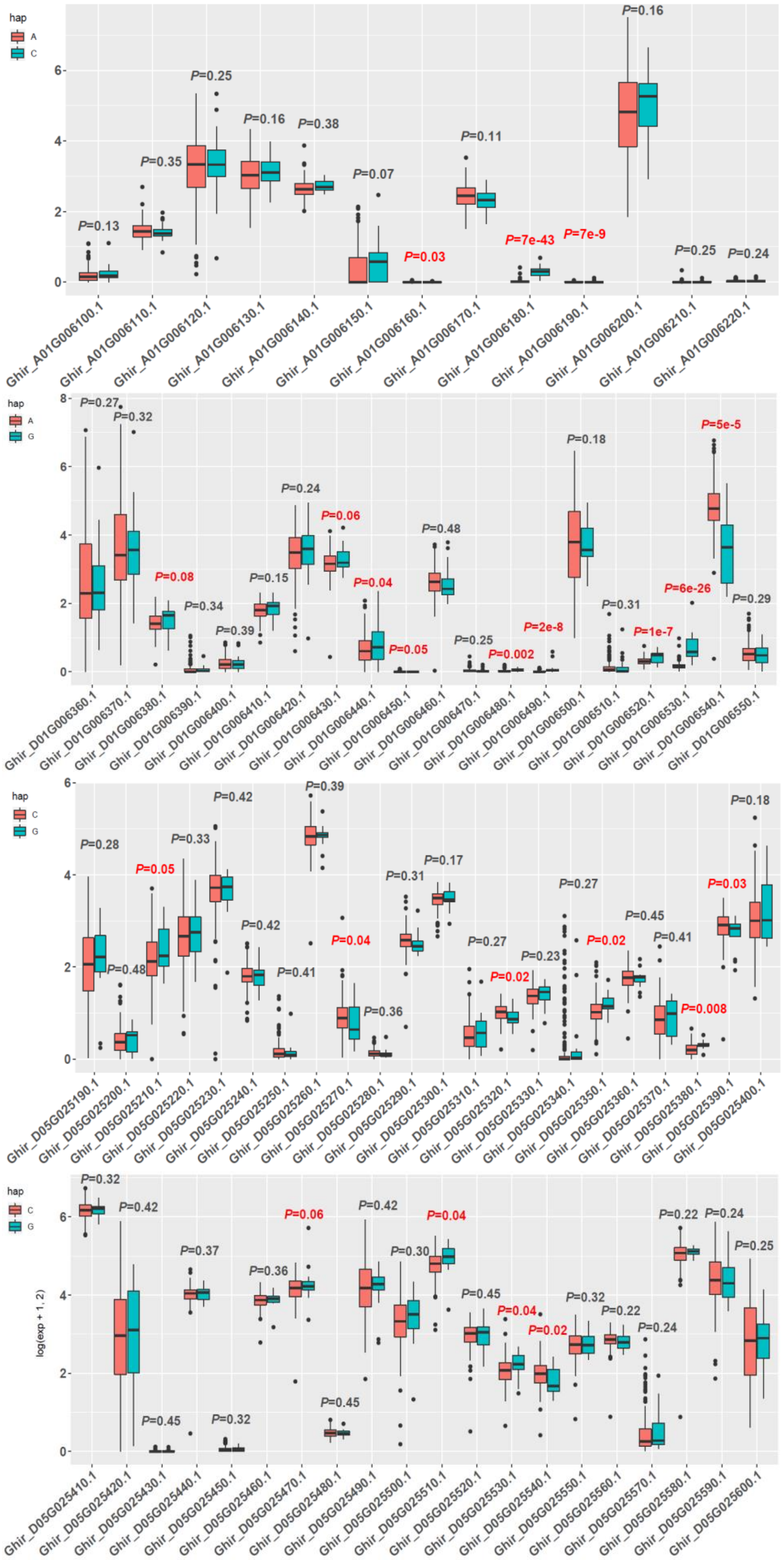

Fig. S13 Transcriptome-wide association study based on express imputation with *cis*-SNPs.

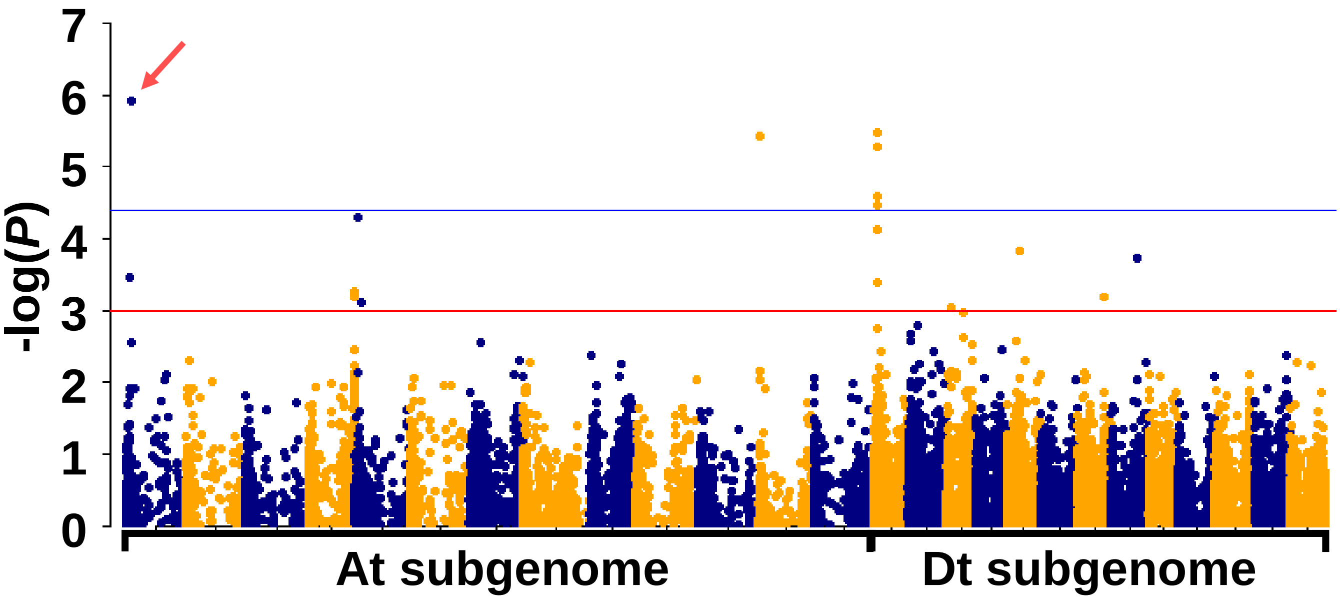

Manhattan plot showing the association between gene expression and pollen viability under high temperature. The highest association signal (salmon arrow) indicated *GhHRK1* identified by GWAS. The blue line represents $P = 1.00\times10^{-3}$ and the red line indicated the Bonferroni-adjusted significance threshold $P = 4.1\times10^{-5}$. Totally 24,338 genes were used to perform TWAS.

Fig. S14 Association analysis in significant intervals in D01 **(a)** and D05 **(b)** chromosome. The detailed linkage disequilibrium and significantly associated genes were provided.

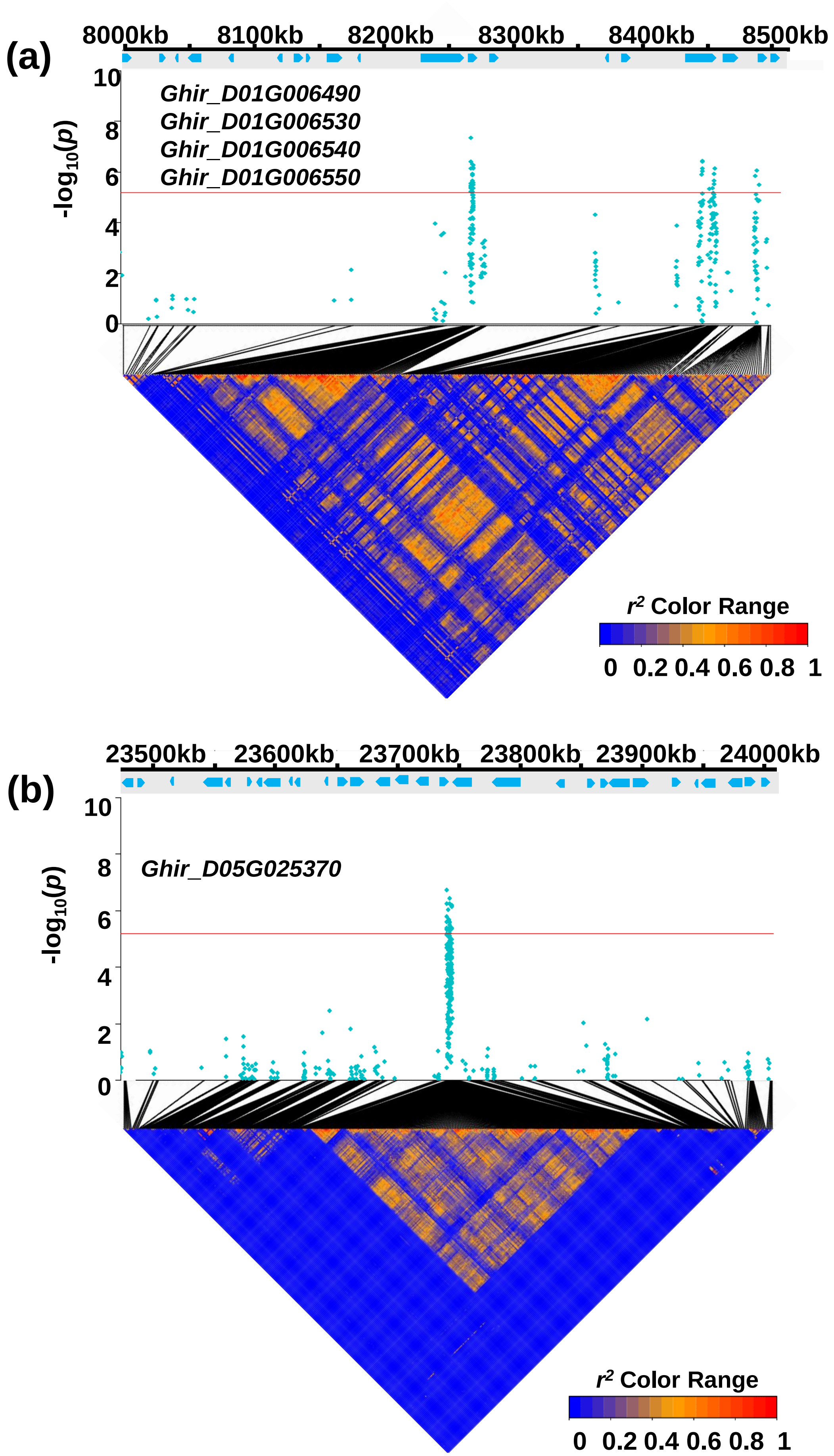

Fig. S15 Protein domain analysis of *Ghir_A01G006180*, *At4g27290* and *GH_A01G0682*.

(a) *Ghir_A01G006180*

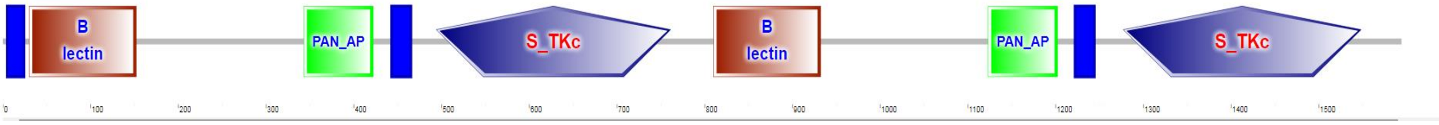

(b) *At4g27290*

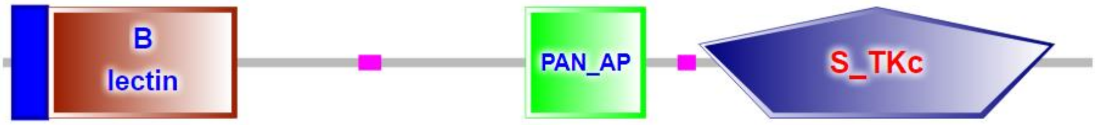

Domain name	Start	End	E-value
B_lectin	30	154	3.35e-35
PAN_AP	344	423	1.45e-15
S_TKc	458	692	7.69e-34

(c) *GH_A01G0682*

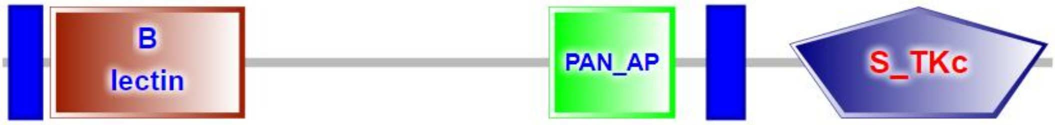

Domain name	Start	End	E-value
B_lectin	30	152	9.05e-41
PAN_AP	343	422	0.0000116
S_TKc	494	656	9.34e-11

(a) The *Ghir_A01G006180* harbored duplicated domains while compared to *GH_A01G0682* and *At4g27290*.
(b) and **(c)** *At4g27290* and *GH_A01G0682* had similar protein domain structures. The table below showed the identical domains and E-value according to SMART protein domain analysis tools. The abbreviations denoted Bulb-type mannose-specific lectin (B_lectin), Apple-like domains (PAN_AP) and Serine/Threonine protein kinases, catalytic domain (S_TKc).

Fig. S16 The coverage of sequencing reads for Ghir_A01G006180 in eight accessions with different phenotypes.

Reads only covered in the first 7 exons of *Ghir_A01G006180*. The salmon arrows showed the fragments for qRT-PCR and *in situ* hybridization.

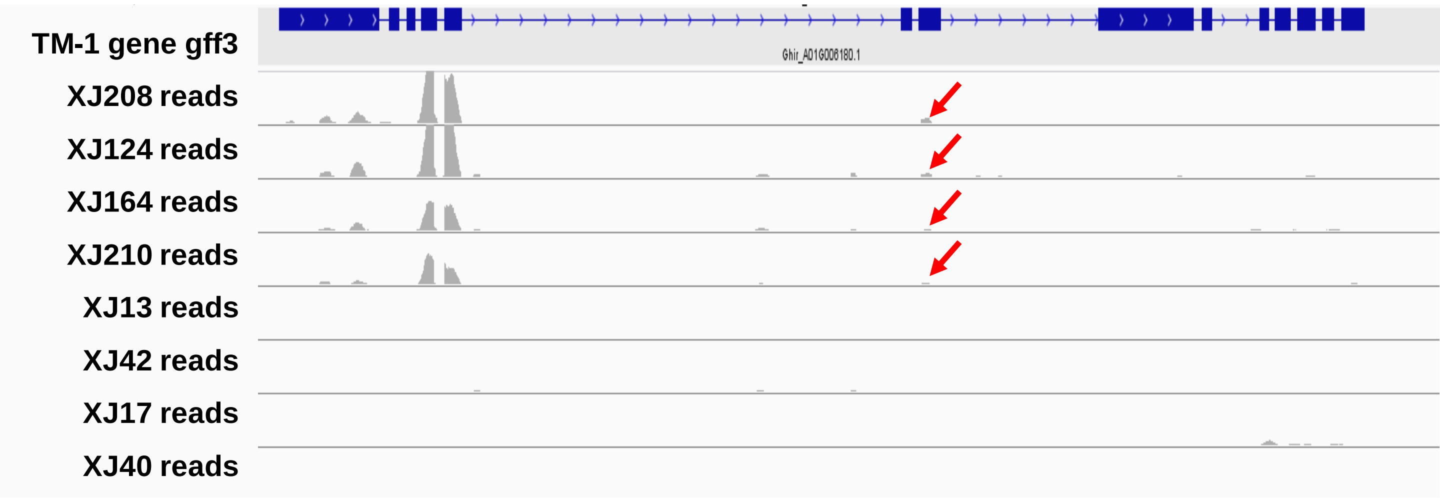

Fig. S17 Nucleic acid sequence alignment of *Ghir_A01G006180*, *GH_A01G0682* and *GH_A01G0683*.

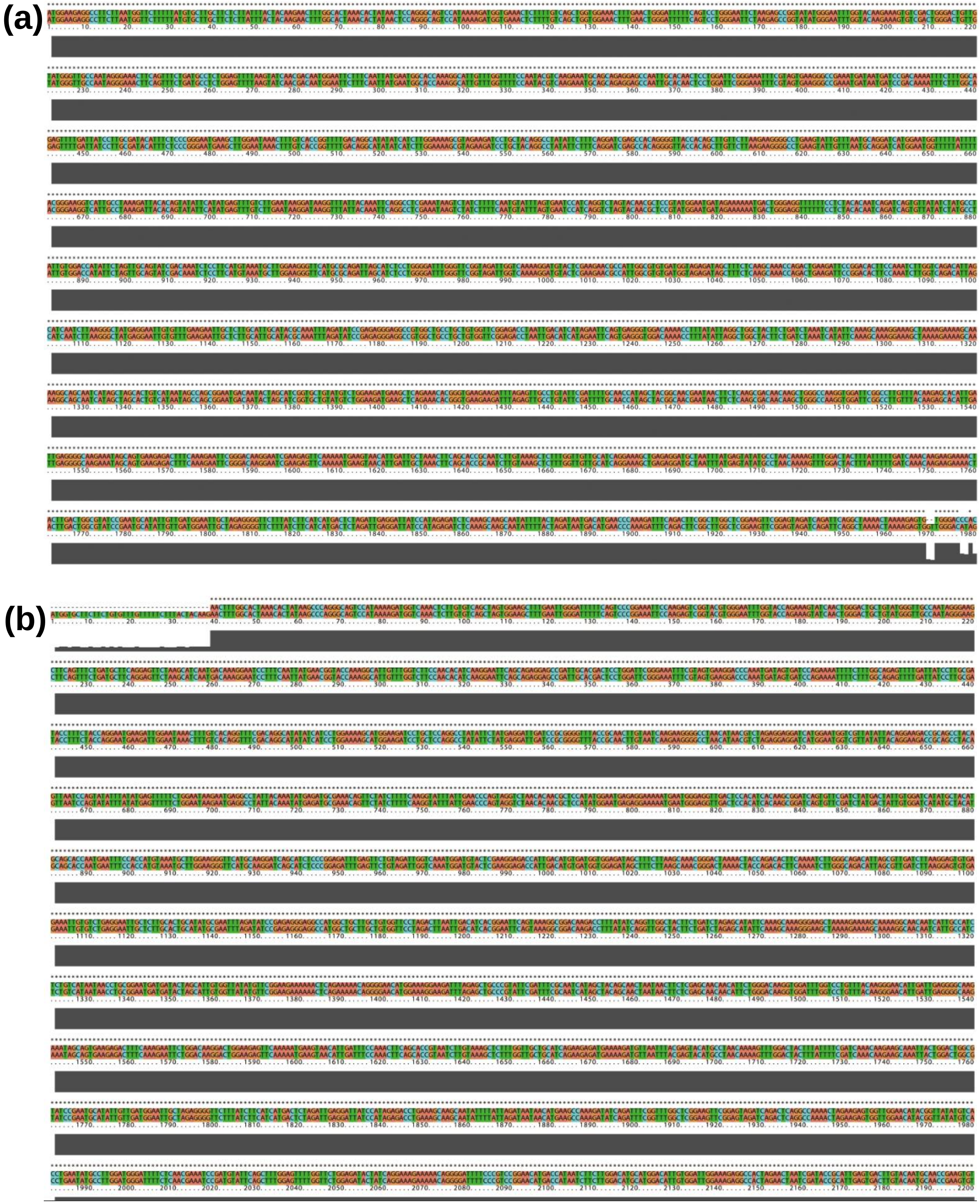

(a) The sequence alignment between the first 7 exons of *Ghir_A01G006180* and *GH_A01G0682*.

(b) The sequence alignment between the last 7 exons of *Ghir_A01G006180* and *GH_A01G0683*.

GH_A01G0682 was estimated to be the casual gene *GhHRK1*.

Fig. S18 Functional annotation of 13 significantly associated SNPs in *GhHRK1*. Nine SNPs were located in the 3' downstream region of *GhHRK1*, one SNP positioned at intron between 2nd and 3rd exons, the rest of three SNPs could induce nonsynonymous mutation.

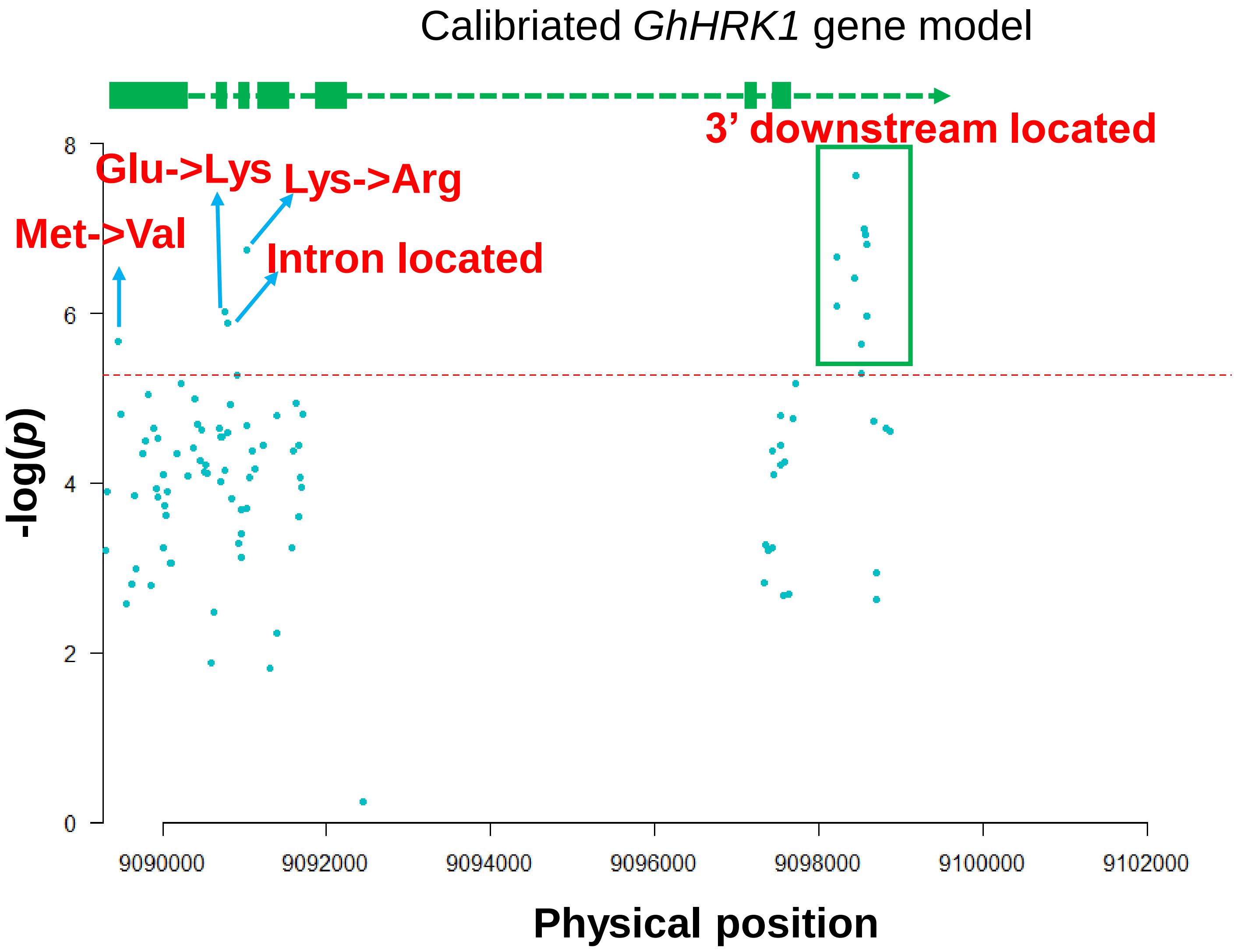

Fig. S19 Differentially expressed MYB transcription factors in accessions with distinct phenotype. The accessions with low pollen viability showed relatively higher expression levels of these MYB transcription factors. The *y*-axis indicated the FPKM values of corresponding genes.

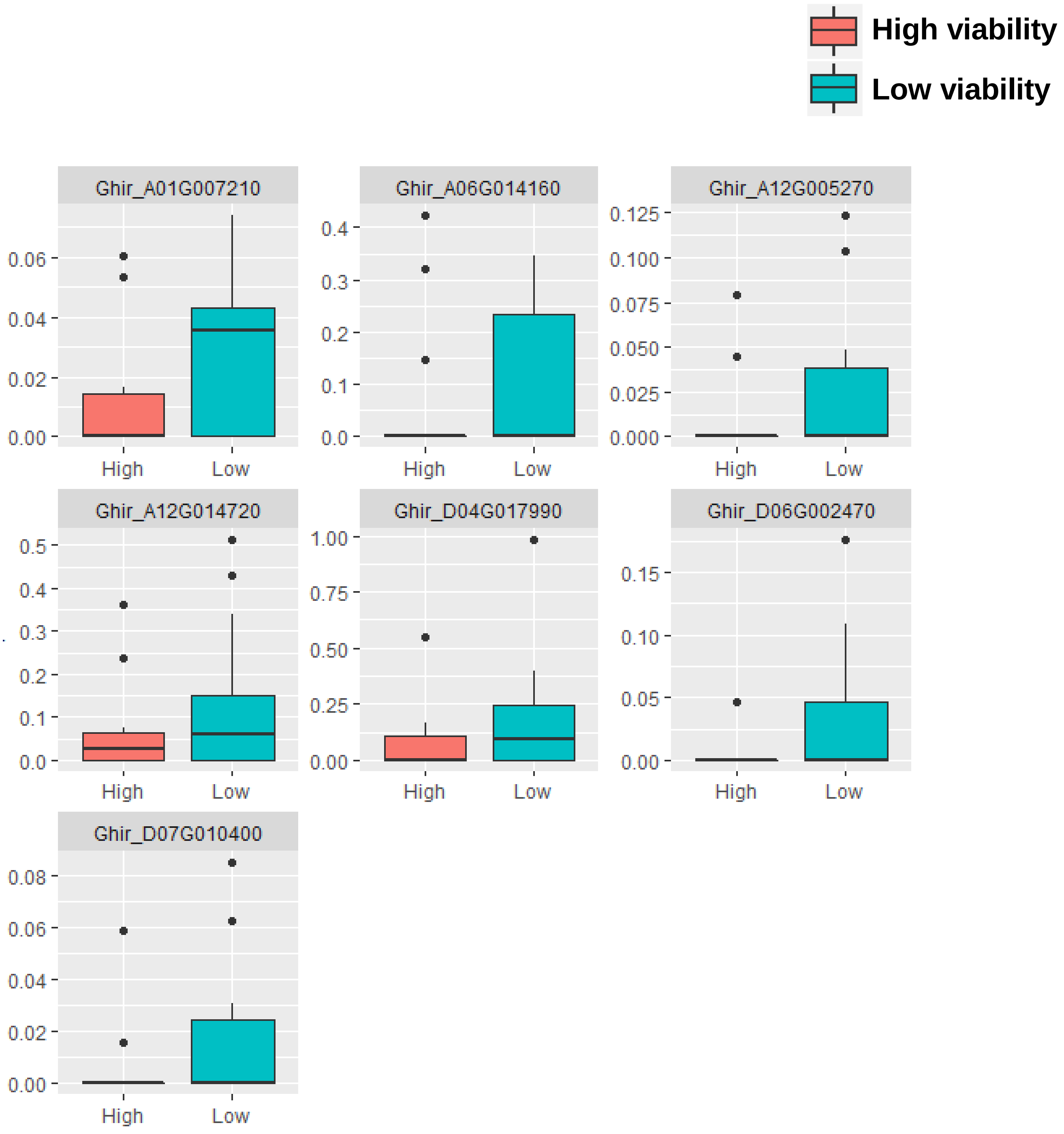

Fig. S20 Pollen viability images of accessions selected to perform *in situ* hybridization. The arrows denoted the sterile pollen grains. Scale = 50 μm.

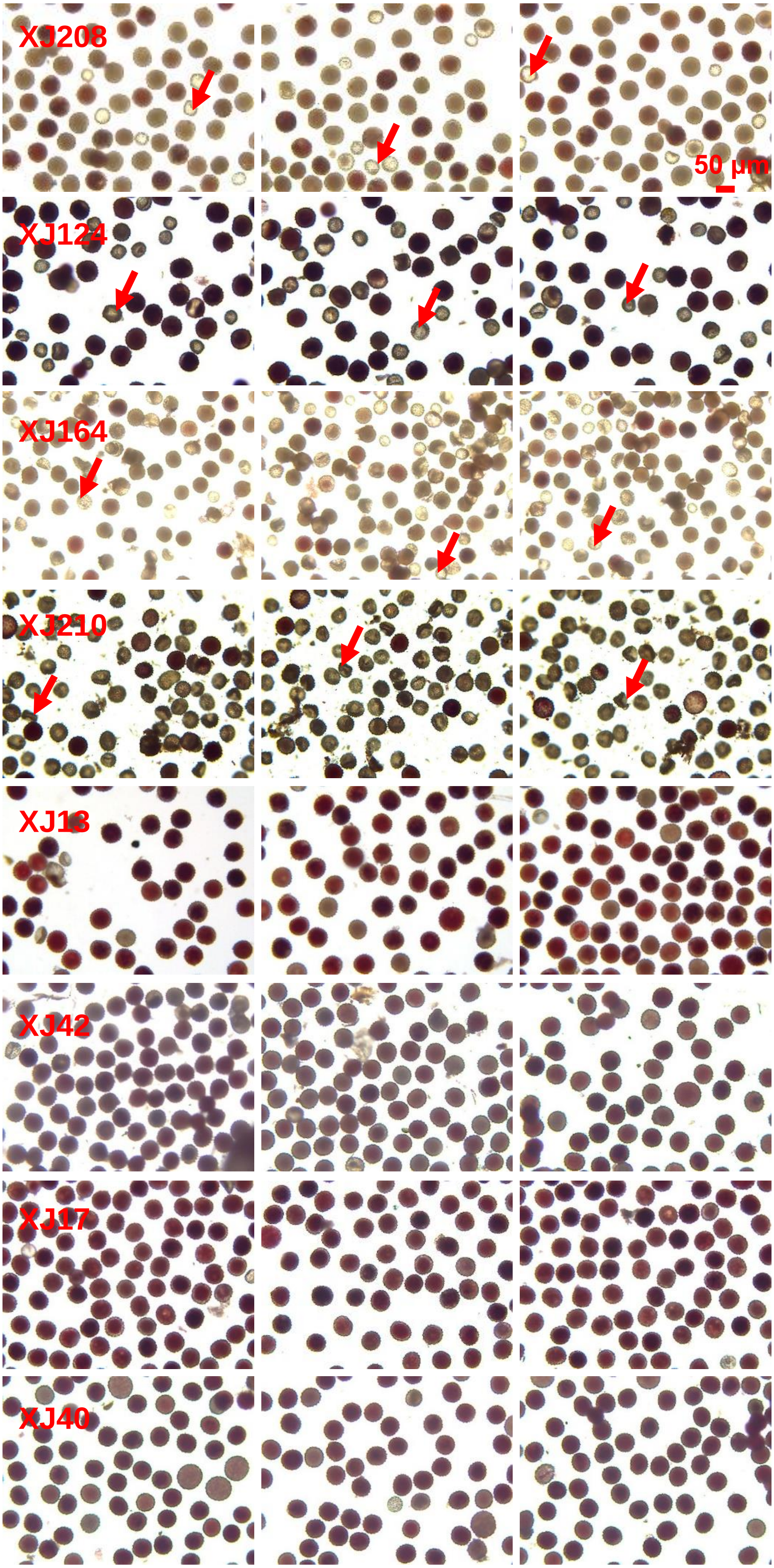

Fig. S21. *In situ* hybridization of *GhHRK1* at tapetum degradation stages of same accessions in Fig. 5.

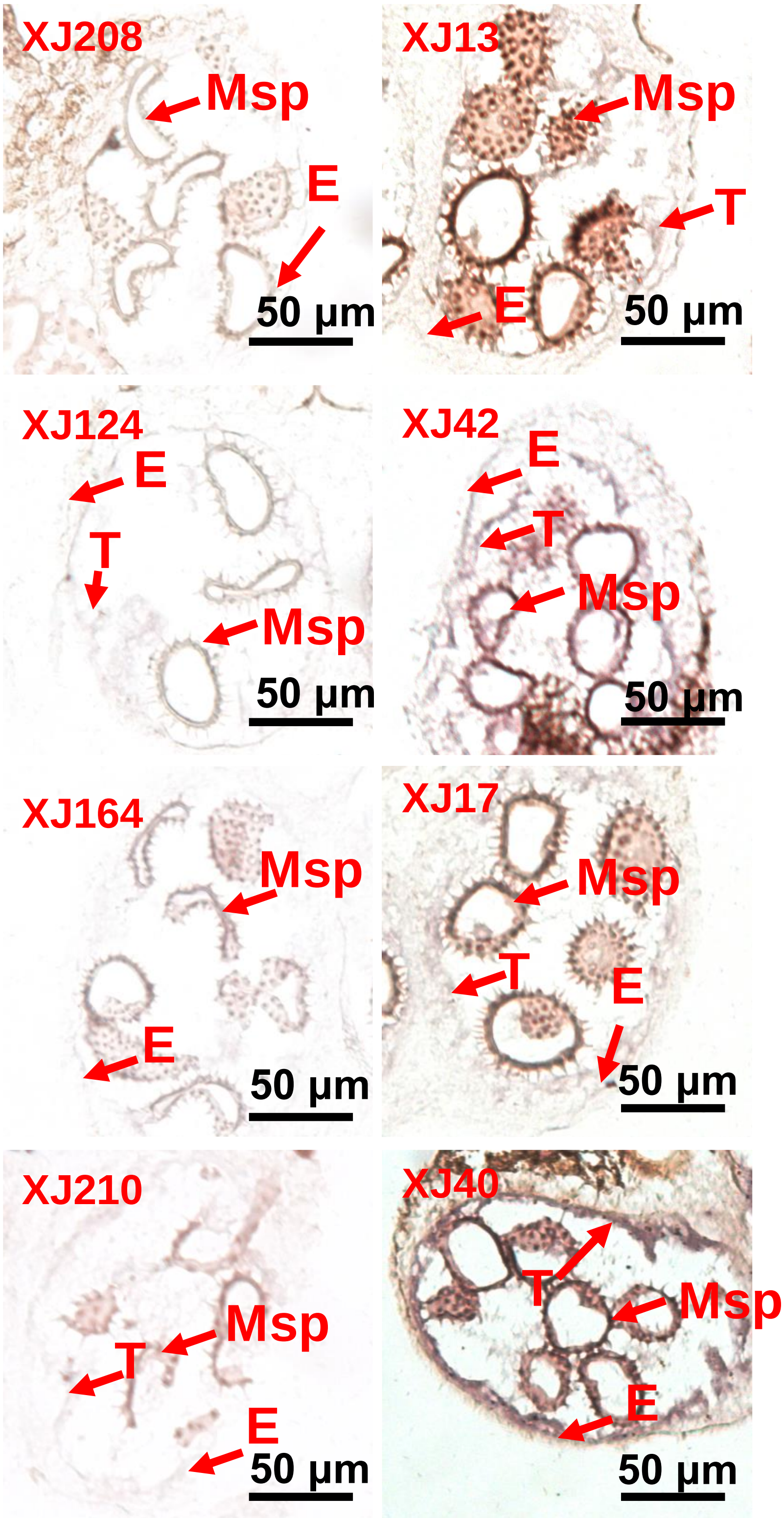

The accessions with increased *GhHRK1* at the tetrad stage showed shriveled microspores and low expression levels in the microspores and tapetum. The accessions with better viability had normal microspore shape and high expression of GhHRK1. XJ208, XJ124, XJ162 and XJ210 were 4 HT-sensitive accessions; XJ13, XJ42, XJ17 and XJ40 were 4 HT-tolerant accessions. T, tapetum; Msp, microspore; E, endothecium. Scale = 50 μm.

Fig. S22. Phylogenic analysis of *GhHRK1* and transcripts of G-type lectin protein kinase in *Arabidospsis*. *At4g27290* was the most adjacent gene to *GhHRK1*.

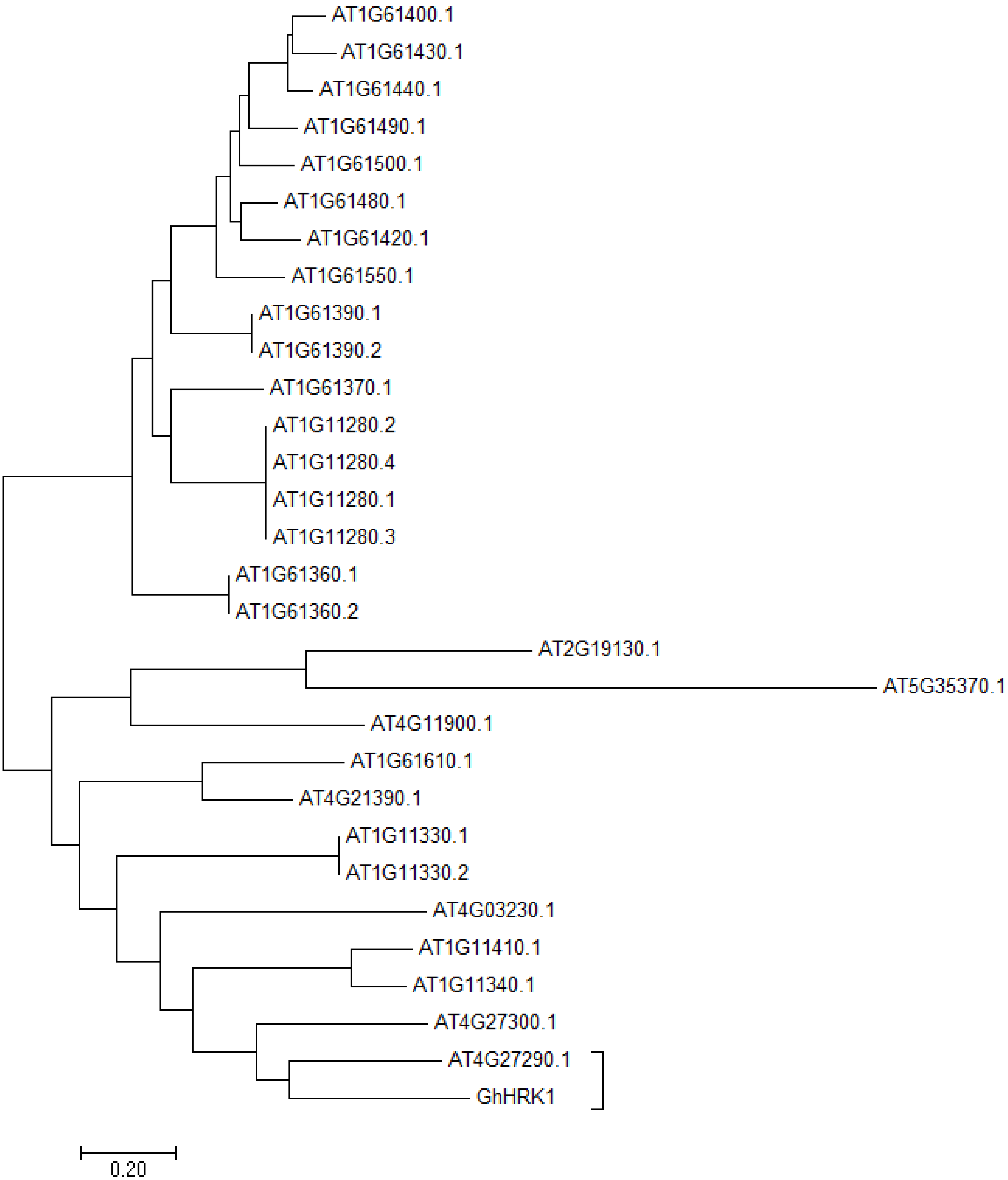

Fig. S23 An overview image of vegetative development of wild type (WT) and two *hrk1* mutant lines during seedling period. Scale = 1 cm.

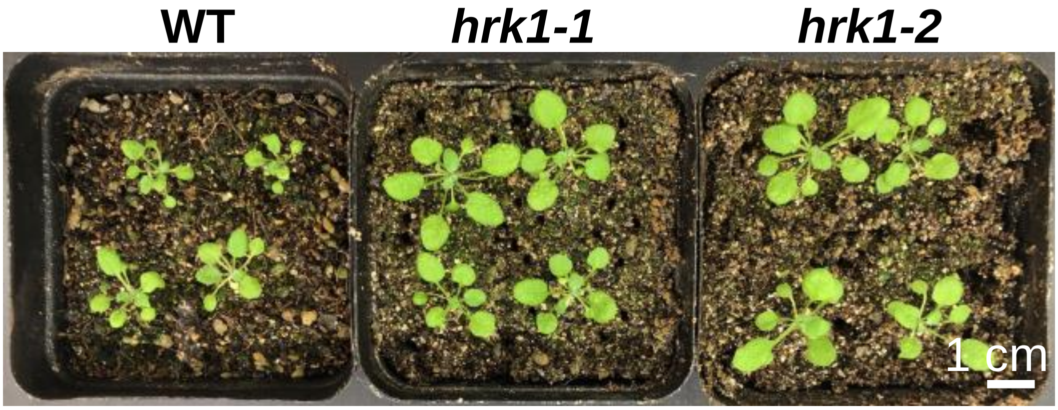

Fig. S24 Pollen viability of WT and two *hrk1* mutants under NT control and HT stress.
After two days treatment of HT stress, WT pollen grains lost viability and those in two mutants still contained relatively normal activity. Normal pollen grains were indicated by salmon arrows while the blue arrows indicated sterile pollen grains. Scale = 100 μm.

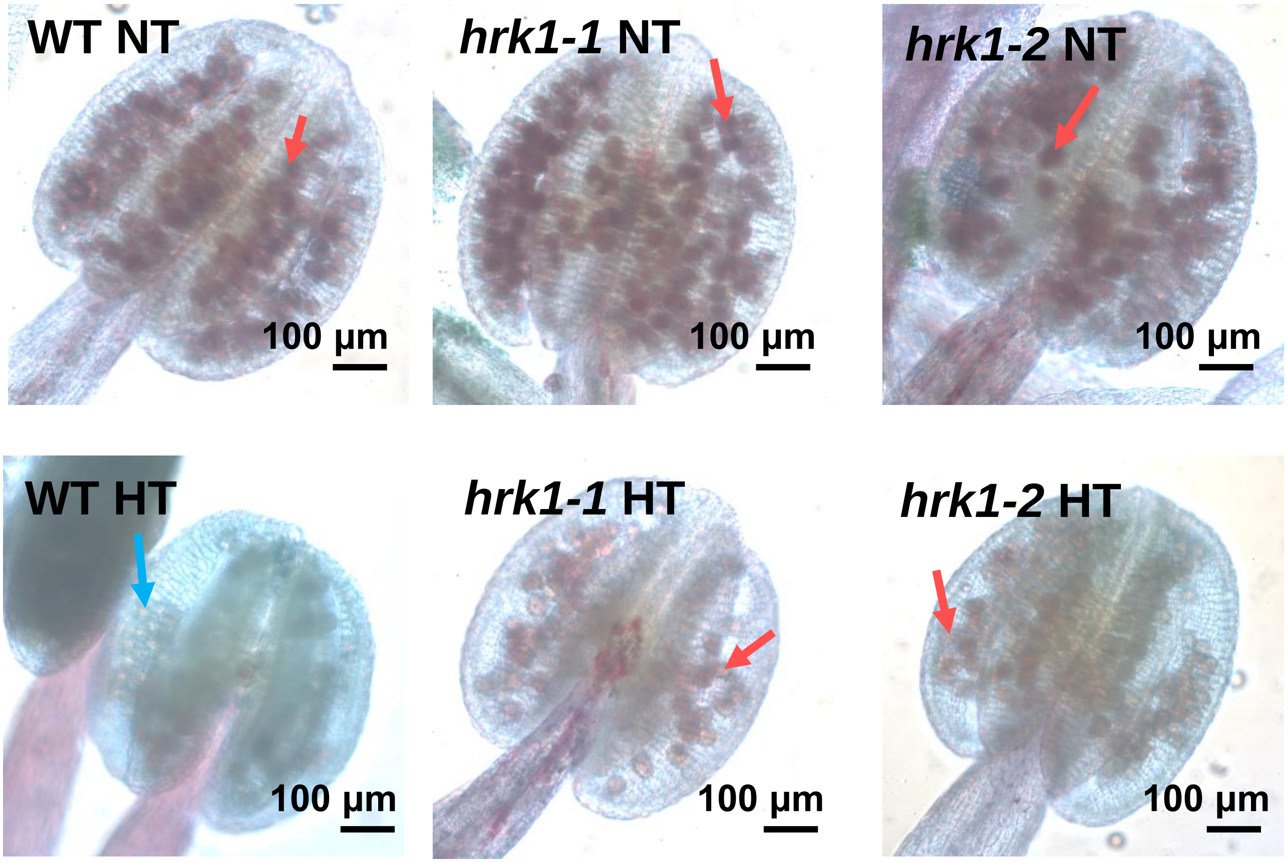

Fig. S25 Images of siliques **(a)** and inflorescence **(b)** of WT, *hrk1-1* and *hrk1-2* after HT treatment. The salmon arrows denoted siliques of WT inflorescence with no seeds. Scale = 1 cm. NT, normal temperature; HT, high temperature.

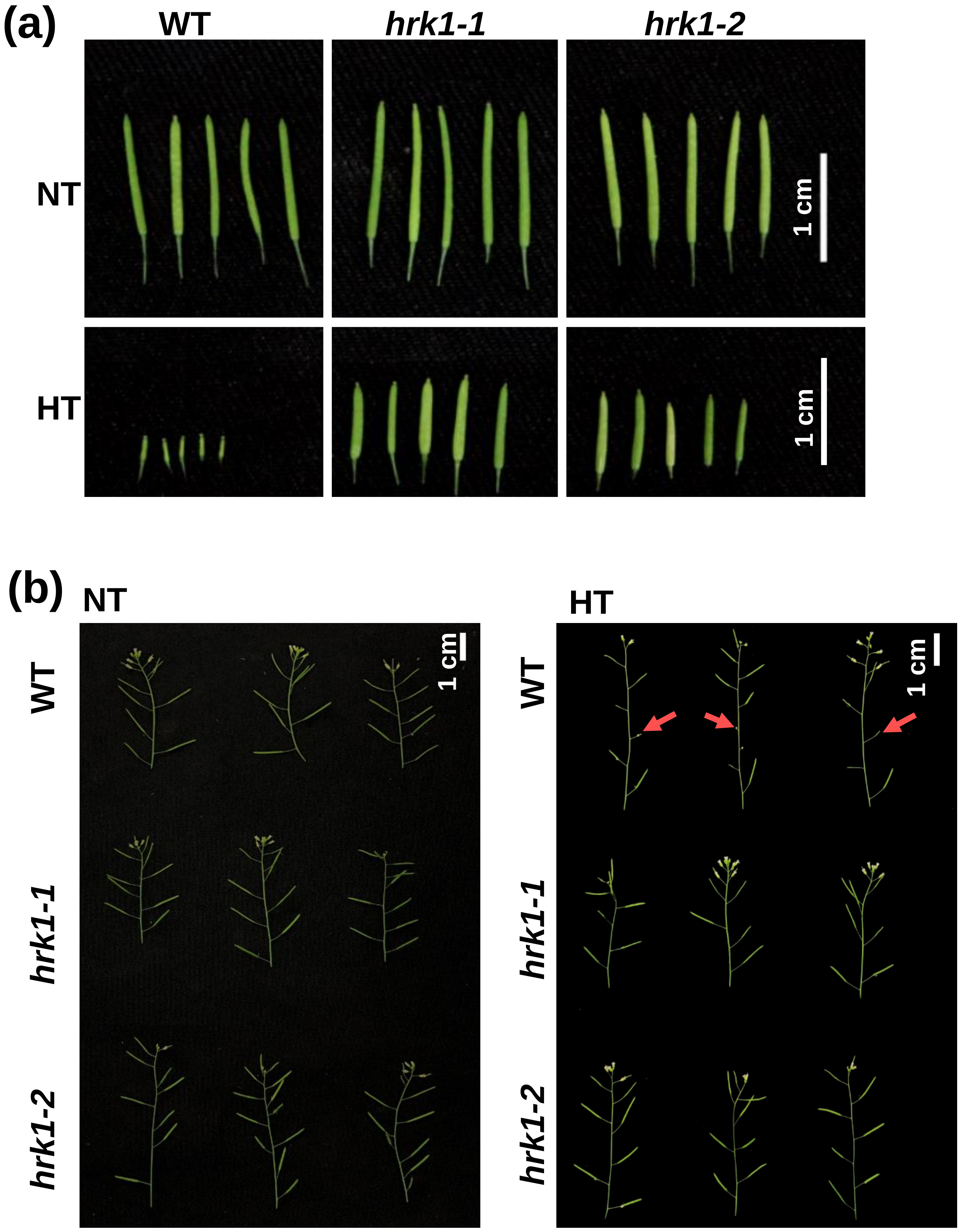

Fig. S26 Transgenic complement assay of *GhHRK1* in *Arabidopsis*. *AtACTIN2* was set as internal control. Three independent transgenic lines were obtained for WT, *hrk1-1* and *hrk1-2* respectively.

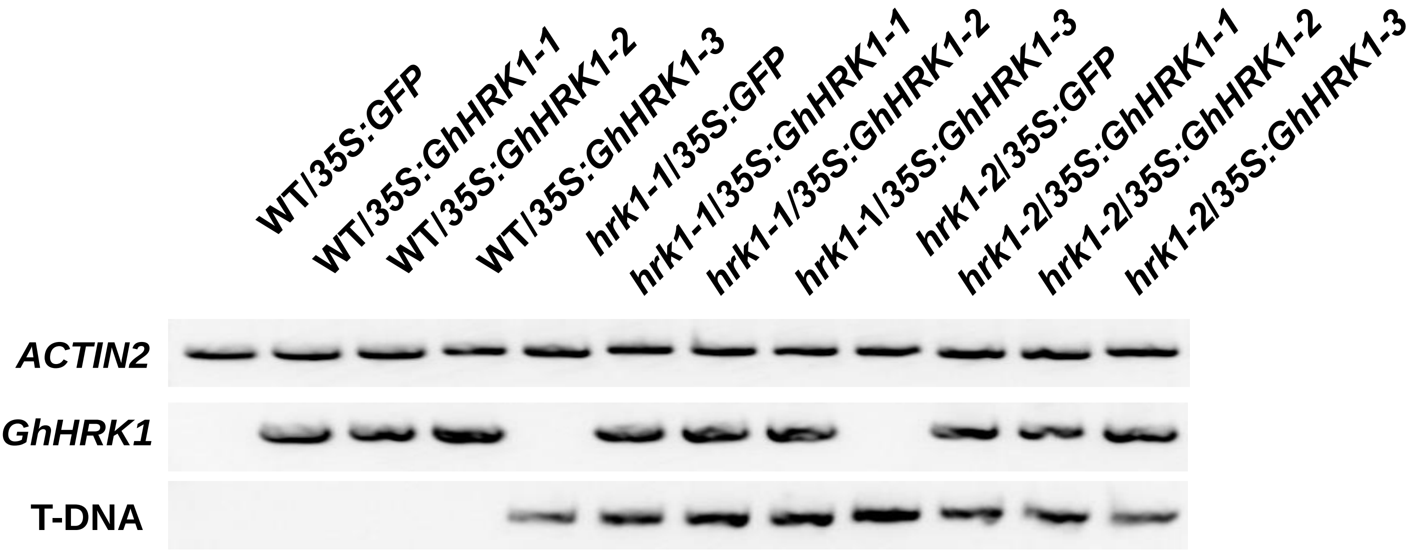

Fig. S27 Phenotypic analysis of *GhHRK1* transgenic individuals NT **(a)** and HT **(b)**.
(a) Low pollen viability was found in WT and mutants expressing *GhHRK1* under NT. NT, normal temperature. Scale = 100 μm.
(b) Individuals expressing *GhHRK1* showed severe male sterility under HT while two mutant lines expressing *GFP* showed relatively normal pollen viability. HT, high temperature. Scale = 100 μm.
The salmon arrows denoted normal pollen grains and the blue arrows denoted aborted pollen grains.

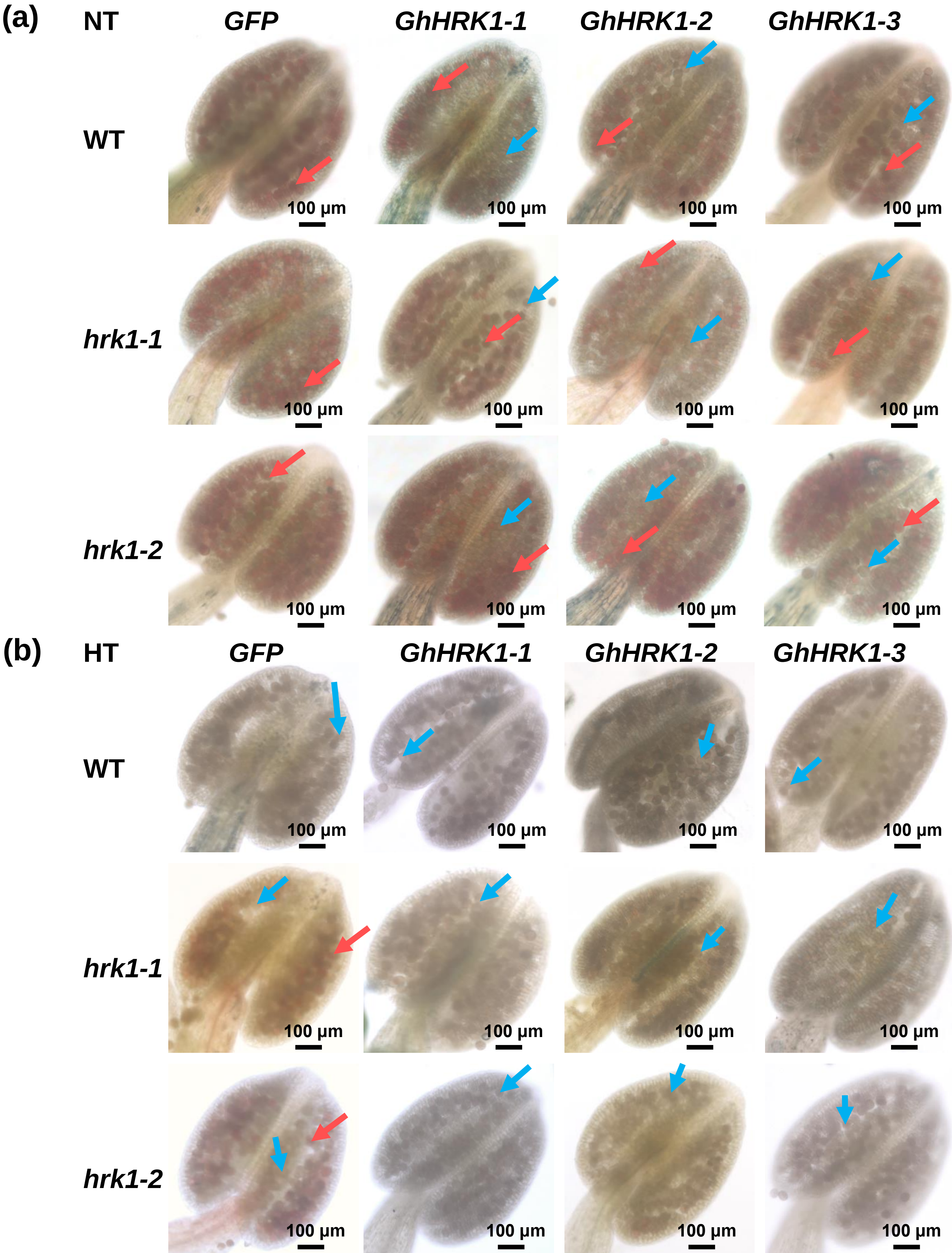

Supplement: Supplementary file 1 — Fig. S1 Images of the experimental fields in Alear, Wuhan, Turpan and the glasshouse in Wuhan. Fig. S2 Analysis of the positions and potential functions of single nucleotide polymorphisms (SNPs). Fig. S3 Analysis of Evanno’s △K from the structure results. Fig. S4 Detailed information of the principal component analysis (PCA) of three subpopulations. Fig. S5 Linkage disequilibrium decay rate in At and Dt subgenomes. Fig. S6 Hierarchical clustering of all accessions. Fig. S7 Proportions of different kinds of transposable element (TE) in the genome. Fig. S8 Analysis of network topology using different soft‐thresholding powers. Fig. S9 Module classification and correlation analysis of each module. Fig. S10 Heatmap of expression profile of 15 modules. Fig. S11 Gene ontology (GO) analysis of genes in the ‘black’ module. Fig. S12 Expression levels of putative associated genes in different genotype accessions. Fig. S13 Transcriptome‐wide association study based on expression imputation with cis‐SNPs. Fig. S14 Association analysis in significant intervals in D01 and D05 chromosomes. Fig. S15 Protein domain analysis of Ghir_A01G006180, At4g27290 and GH_A01G0682. Fig. S16 The coverage of sequencing reads for Ghir_A01G006180 in eight accessions with different phenotypes. Fig. S17 Nucleic acid sequence alignment of Ghir_A01G006180, GH_A01G0682 and GH_A01G0683. Fig. S18 Functional annotation of 13 significantly associated SNPs in GhHRK1. Fig. S19 Differentially expressed MYB transcription factors in accessions with distinct phenotypes. Fig. S20 Pollen viability images of accessions selected for in situ hybridization. Fig. S21 In situ hybridization of GhHRK1 at the tapetum degradation stage for the same accessions as in Fig. 5. Fig. S22 Phylogenic analysis of GhHRK1 and transcripts of G‐type lectin protein kinase in Arabidopsis. Fig. S23 An overview image of vegetative development of the wild‐type (WT) and two hrk1 mutant lines during the seedling period. Fig. S24 Pollen viability [file NPH-231-165-s002.pdf]
